# Occurrence and characterization of tremolite asbestos from the Mid Atlantic Ridge

**DOI:** 10.1038/s41598-021-85576-w

**Published:** 2021-03-18

**Authors:** Dario Di Giuseppe, Natale Perchiazzi, Daniele Brunelli, Tommaso Giovanardi, Luca Nodari, Giancarlo Della Ventura, Daniele Malferrari, Marcia Maia, Alessandro F. Gualtieri

**Affiliations:** 1 Department of Chemical and Geological Sciences, University of Modena and Reggio Emilia, Modena, Italy; 2 Department of Sciences and Methods for Engineering, University of Modena and Reggio Emilia, Reggio Emilia, Italy; 3 Department of Earth Sciences, University of Pisa, Pisa, Italy; 4 CNR-ISMAR Institute for Marine Sciences, Italian National Research Council, Bologna, Italy; 5 CNR-ICMATE Institute of Condensed Matter Chemistry and Technologies for Energy, Italian National Research Council, Padua, Italy; 6 Department of Sciences, University of Roma Tre, Rome, Italy; 7 INFN Laboratori Nazionali di Frascati, Frascati (Rome), Italy; 8 CNRS- Géosciences Océan UMR 6538 - Institut Universitaire Européen de la Mer, Plouzané, France

**Keywords:** Solid Earth sciences, Mineralogy

## Abstract

Tremolite is one of the most common amphibole species and, in the fibrous form (i.e., characterized by crystals/particles consisting of fibres with length > 5 µm, width < 3 µm and aspect ratio > 3), one of the six asbestos minerals. Until now the attention of crystallographers has focused only on samples from continental environment. Here we report the first chemical and structural data of a tremolite asbestos found along the Mid Atlantic Ridge (MAR) at the eastern intersection of the Romanche Transform Fault (Equatorial MAR). Tremolite is associated with chlorite and lizardite and was formed through the green shale facies lower than zeolite in a predominantly fluid system. MAR tremolite asbestos shows very slight deviations from the ideal crystal structure of tremolite. Differences in cation site partitioning were found with respect to tremolite asbestos from ophiolitic complexes, attributed to the different chemical–physical conditions during the mineral formation. In particular, oceanic tremolite asbestos is enriched in Al and Na, forming a trend clearly distinct from the continental tremolites.

## Introduction

Asbestos is a general term that collectively refers to six mineral species: chrysotile and five fibrous amphiboles of commercial interest^1–3^. The family of amphibole asbestos includes: amosite (fibrous variety of grunerite), crocidolite (fibrous variety of riebeckite), fibrous actinolite, fibrous anthophyllite and fibrous tremolite^1–3^. Chrysotile is the product of the serpentinization of ultramafic rocks (dunite, peridotite, and pyroxenite) and is present in low-grade serpentinites from the oceanic lithosphere and from low-grade metamorphic ophiolites^2,4,5^. The most valuable deposits of chrysotile occur in continental ophiolite massifs^2,5^ and the main chrysotile asbestos mines being located in Urals (Russian Federation) and the Appalachians (USA and Canada)^2^. The major world deposits of crocidolite and amosite are located within the Precambrian banded iron formations (BIF) in southern Africa and western Australia^2,6^. BIF are chemical sedimentary rocks characterized by alternating layers of Fe-rich minerals and microcrystalline quartz^6,7^. It is generally recognized that crocidolite and amosite developed within the BIF under low T/P metamorphic conditions^6,7^. Tremolite, actinolite, and anthophyllite asbestos occur in serpentinized basic to ultra-basic igneous rocks commonly found in ophiolitic complexes which represent the main host formations for these minerals^2,4,5^.

Although fibrous minerals are widespread in the oceanic lithosphere^8,9^, the human exposition to mineral fibres has naturally confined the study of these species to the continental occurrences^2,3^. However, it is of great meaning exploring the compositional evolution and diversity of the oceanic and continental domains, being the oceanic lithosphere the main precursor of the continental deposits. For instance, while chrysotile is a normal occurrence in continental formations, in the oceanic domain, the pervasive serpentinization of the ultramafic rocks is largely dominated by the pseudomorphic crystallization of lizardite + magnetite (± brucite) at the expenses of olivine and pyroxenes^9^, being chrysotile only a minor occurrence restricted to late vein assemblages^8^.

Here, we focus on the occurrence of tremolite that, along with hornblende and pargasite is among the most abundant amphibole phases reported from the Mid Ocean Ridges^8–12^. Tremolite is a monoclinic (*C*2/*m*) calcic amphibole with ideal formula Ca_2_Mg_5_Si_8_O_22_(OH)_2_ that forms a solid solution series with actinolite (Ca_2_(Mg,Fe^2+^)_5_Si_8_O_22_(OH)_2_) and ferro-actinolite (Ca_2_(Fe^2+^,Mg)_5_Si_8_O_22_(OH)_2_)^4^. Tremolite-actinolite, both in veins and groundmass have been described in serpentinized rocks from the MAR associated to shear zones, melt injection a pervasive high-T weathering processes^8–12^. Despite a large abundance of compositional and petrological analyses, a characterization of the crystal structure and crystal chemistry of tremolite and tremolite asbestos from MAR have not been reported yet.

Hereafter the term “fibre” is used to indicate an elongate mineral particle, eventually forming fibre bundles, with length (L) > 5 µm and width (W) < 3 µm and aspect ratio (L/W) > 3; “fibrous” refers to mineral particles that occur as fibres; “fibre bundle” is a group of parallel fibres bound together; “elongate mineral particle” is a particle with and aspect ratio > 3 and a “cleavage fragment” is a particle that may have the same chemical composition of the asbestos or asbestiform counterparts but that cleaves into fragments rather than separate longitudinally into a fibre. Because tremolite is one of the six asbestos species, “tremolite asbestos” is defined as the fibrous variety of tremolite^3,13,14^.

**Figure 1:**
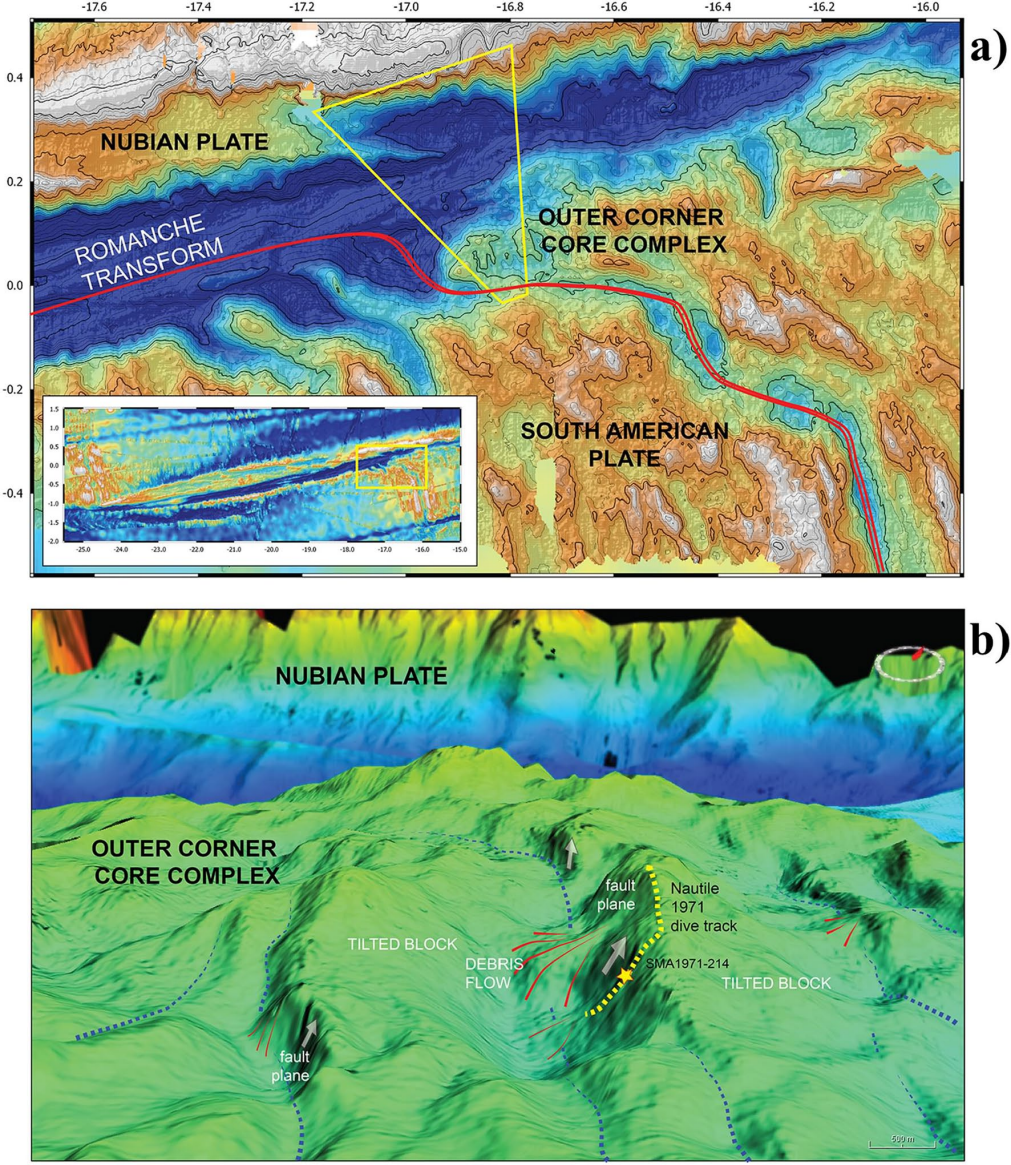
(**a**) Bathymetry of the eastern intersection of the Romanche Fracture with the Mid Atlantic Ridge, (**b**) 3-D reconstruction of the area in (yellow region in **a**). Topographic features have been observed during the Nautile dive SMA1971 in the oceanic cruise SMARTIES, 2019. Position of sample SMA1971-214 (yellow star) is reported along the Nautile dive track (yellow dot line). Bathymetry and 3D rendering have been generated from shipborne multibeam echosounding data acquired during the SMARTIES cruise^15^. Data were acquired with a Reson Seabat 7150 echosounder, using 880 beams at 12/24 kHz with an average swath of 12 km. Data were processed with the Globe software by IFREMER^65^.

Recently (July–August 2019) a joint French-Italian expedition to the Equatorial MAR (SMARTIES) explored the eastern intersection of the Romanche Transform Fault with the MAR^15^ (Fig. 1). Numerous samples have been recovered at extreme depths by manned submersible exploration, reaching and encompassing 6 km b.s.l. Glassy basalts were recovered from a narrow magmatic region while plagioclase-bearing peridotites and ultramafic ultramylonites were recovered from a large tectonic dome forming an oceanic core complex on the outer corner of the ridge-transform intersection (Fig. 1). These mantle-derived ultramafic rocks are in general largely serpentinized and tectonized. Among those samples an extraordinary occurrence of a greenish amphibole mineralization was recovered (SMA1971-214); it is made of well-developed and unaltered tremolite sample. This sample was taken from the surface of a normal fault, possibly representing a slickenside mineralization related to its displacement (Supplementary Fig. S1).

In this study we report the first detailed morphological and crystal-chemical characterization of tremolite asbestos from the oceanic lithosphere. The chemical composition of SMA1971-214 tremolite sample was obtained by electron probe micro analysis (EPMA). Crystal structure refinement was modelled by combining chemical, spectroscopy and X-ray single-crystal diffraction data. Scanning electron microscope (SEM) and transmission electron microscopy (TEM) data were combined and integrated, to characterize the morphology of the mineral. Thermal behaviour was described through thermo-differential and thermo-gravimetric measurements, the latter coupled with evolved gasse mass spectrometry.

## Results

**Figure 2:**
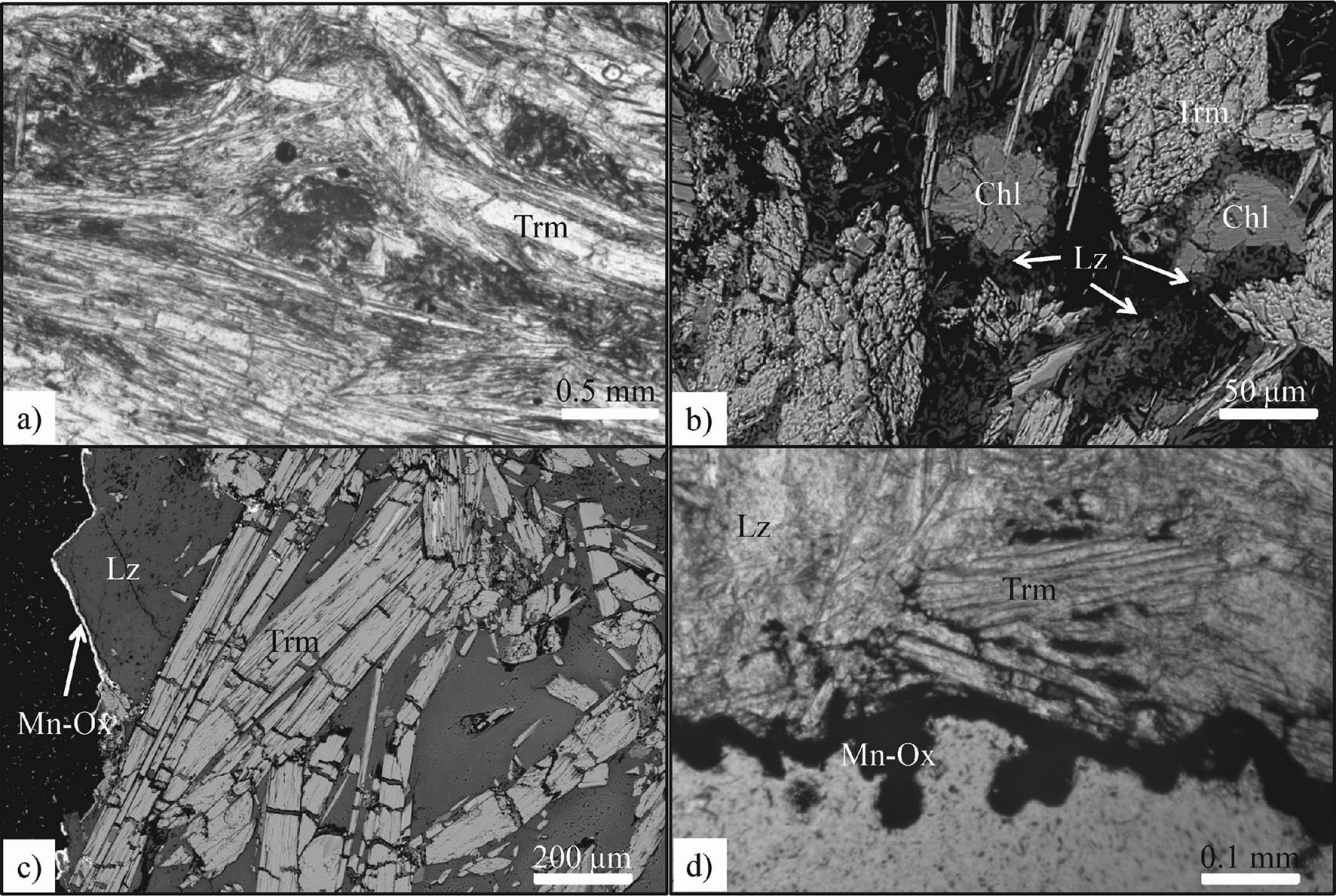
(**a**) Sample SMA1971-214 observed with polarized light optical microscopy (plane-polarized light). Tremolite (Tmr) fibres with orientation are grouped in sheaf-like clusters: the darkest regions are mainly basal sections while lighter ones are lateral sections. Detail of folded and kinked tremolite by perpendicular overgrowth of another bundle. (**b**) SEM image of tremolite (Tmr), secondary rounded chlorite (Chl) and late lizardite (Lz) around crystals and filling fractures. (**c**) SEM image of late lizardite (Lz) domain with tremolite (Tmr) fibres at the border of the sample coated by Mn-oxides (Mn-Ox). (**d**) The Mn-oxides (Mn-Ox) coating envelops the sample and spreads within.

**Figure 3:**
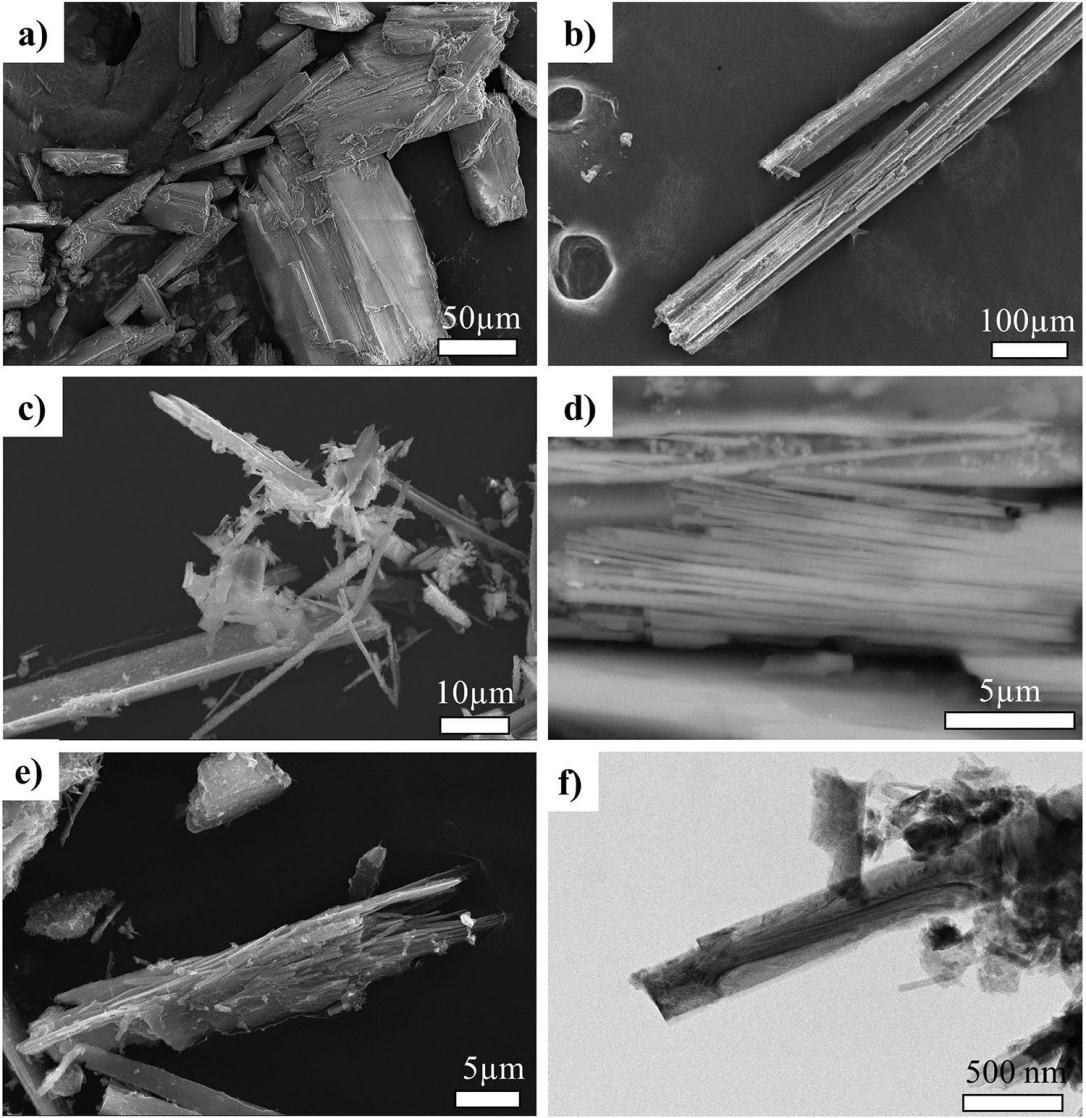
SEM and TEM images of tremolite find in the sample SMA1971-214. (**a**) SEM images of columnar aggregates with elongated prismatic habit. (**b**) SEM image of fibres bundles. (**c**) Tremolite asbestos fibres, (**d**) SEM images of tremolite fibres bundles. (**e**) SEM images of bundle of thin tremolite fibres, (**f**) TEM images of the tremolite single fibre and encrusting lizardite.

Sample SMA1971-214 has been collected from the surface of a normal fault in the Eastern Ridge-Transform Intersection of the Romanche Transform Fault recovered during the dive SMA1971 at 3978 m b.s.l.^15^ (Fig. 1 and Supplementary Fig. S1). It appears as a block of greenish crystals coated by a thin manganese crust (Fig. 2). The manganese coating covers the outer amphibole crystals for ≤ 0.2 mm (Fig. 2c,d) attesting for a recent exposure to the seafloor. Electron and optical microscopy investigations show that the sample is mainly formed by tremolite that occurs with two main crystal habits: elongated prismatic, including elongate mineral particles and cleavage fragments (major) and fibrous (subordinate) (Figs. 2, 3). Tremolite asbestos exhibits a wavy structure overprinted by late kinking and folding, suggesting a polyphasic deformation following the primary sin-kinematic crystallization (Fig. 2a,b,c). Pressure-shadows associated to sigmoidal tremolite structures are filled by chlorite and serpentine (Fig. 2a and Supplementary Fig. S2). Observed fibres (Fig. 3 and Supplementary Fig. S3) have lengths ranging 5.12–93.4 μm (average 22.3 μm) and widths between 0.17 and 3.03 μm (average 1.17 μm) (see Supplementary Table S1 for the complete dataset). All of the observed fibres have aspect ratio L/W > 3 (average 24.6). Chlorite appears as round-shaped crystals, developed in the interstices or near fractures (Fig. 2b). Lizardite fills late fractures and the interstices of both tremolite and chlorite crystals (Fig. 2b,c; Supplementary Information), locally englobing few large tremolite crystals (Figs. 2c, 3d and Supplementary Fig. S3). On this base we have recognized the following mineral crystallization sequence: tremolite, chlorite, lizardite and, finally, Mn-Oxides.

Results of the thermo-gravimetric and thermo-differential analyses (TGA-DTA) of SMA1971-214 showed six main thermal events at 35, 594, 726, 785, 834 and 1059 °C (Supplementary Fig. S4a). The first, at 35 °C, is due to the loss of weakly adsorbed water^16^. The reactions at 594, 726 and 785 °C denote the release of bound water (Supplementary Fig. S4b) and are related to the dehydroxylation of chlorite (594 and 785 °C) and lizardite (726 °C)^16–18^. The exothermic reaction with maximum at 834 °C marks the recrystallization into new phases^16–18^. The sharp endothermic peak at 1059 °C corresponds to breakdown of tremolite^16^as shown by the release of H_2_O (Supplementary Fig. S4b). Lack of significant CO_2_, NO, H_2_S, NO_2_ and SO_2_ degassing rules out the occurrence of phases containing thermally removable amounts of these gasse (Supplementary Fig. S4b).

Major element EPMA analyses (Supplementary Table S2) allowed classifying the amphibole from sample SMA1971-214 as tremolite according to Hawthorne et al.^19^. Average ^tot^Mg# value (Mg# = Mg^2+^/(Mg^2+^ + Fe^2+^_tot_) × 100) is 93.91 while ^C^Mg# is 97.00. Results of EMPA spot analyses show homogenous composition on 33 spots (Supplementary Table S2). SiO_2_ is in the range of 52.79–55.76 wt% with an average of 54.21 wt%. MgO is 22.44 wt% in average while CaO is 12.10 wt% in average. Larger variability is shown by Al_2_O_3_ (between 2.78 and 4.27wt%, average = 3.73 wt%) and Cr_2_O_3_ (between 0.07 and 0.90 wt%, average = 0.44 wt%). Minor elements (i.e*.*, Pb, Co, V, Cu, F and Cl) are always close or below the detection limits.

The Mössbauer spectrum of the SMA1971-214 sample, reported in Supplementary Fig. S5, highlighted the typical absorptions for ferrous and ferric paramagnetic species^20^. Given the nature of the analysed sample, these absorptions are due to the Fe nuclei hosted in both accessory and principal phases. Several models with unconstrained and constrained parameters were tested to obtain a reasonable fit. The best result, with the lowest uncertainties on the hyperfine parameters, was obtained by a Quadrupole Splitting Distributions (QSD) approach^21^, consisting in two sites representative for the ferric and ferrous population. The ferric Site 1 is described by a single component whereas the ferrous Site 2, by two components. Site 1 shows parameters typical of ferric in a distorted octahedral environment, and it can be reasonably ascribed to lizardite and chlorite. Concerning Site 2, QSD highlights the presence of a binomial distribution of Δ showing two maxima at 2.73 and 1.79 mm/s respectively. The hyperfine parameters are reported in Supplementary Table S3. The values of 〈Δ〉 are typical of distorted octahedral sites, and they can be attributed to ferrous nuclei contained in tremolite and, eventually, within accessory phases^20,21^. Unfortunately, the impossibility to separate these contributions does not allow a rigorous correlation between the ferrous population and the tremolite crystallographic sites.

The structural formula derived from major element EPMA analyses can be expressed as:

$$\begin{aligned} & \left( {{\text{Na}}_{0.25} {\text{K}}_{0.01} } \right)_{\sum = 0.26} \left( {{\text{Ca}}_{1.80} {\text{Fe}}^{2 + }_{0.17} {\text{Na}}_{0.03} } \right)_{\sum = 2} \\ & \quad \left( {{\text{Mg}}_{4.65} {\text{Al}}_{0.14} {\text{Fe}}^{3 + }_{0.13} {\text{Cr}}_{0.05} {\text{Ti}}_{0.01} {\text{Ni}}_{0.01} {\text{Mn}}_{0.01} } \right)_{\sum = 5} \left( {{\text{Si}}_{7.53} {\text{Al}}_{0.47} } \right)_{\sum = 8} {\text{O}}_{22} ({\text{OH}})_{2} \\ \end{aligned}$$

Cell constants *a* = 9.8469(6) Å, *b* = 18.0651(11) Å, *c* = 5.2795(4) Å, *β* = 104.803(3)°, and V = 907.98(9) Å^3^, derived from structural refinement (Supplementary Table S4), fall quite close to the predicted unit cell parameters *a* = 9.841(3) Å, *b* = 18.055(4) Å, c = 5.278(1) Å, β = 104.72° (2), and V = 906.6(5) Å^3^ reported for end-member tremolite^22^. Values extrapolated for pure tremolite^23^ are: *a* = 9.873(15) Å, *b* = 18.057(11) Å, *c* = 5.268(5) Å, β = 104.94(8)°, V = 907.4 Å^3^. Refined atomic coordinates, selected bond lengths, angles and polyhedral distortion parameters^24^ for SMA1971-214 tremolite asbestos are reported in Supplementary Table S5 and S6. The crystal structure of tremolite asbestos is represented in Supplementary Fig. S6. Anisotropic displacement parameters are reported as Supplementary Information (cif file). Site occupancies derived from the structural refinement (see Table 1) were: Na_0.29_ for the *A* site, Mg_1.92_Fe_0.08_ for *M*(1) and *M*(2) sites, Mg_0.96_Fe_0.04_ for the *M*(3) site, Ca_1.92_ and Fe_0.08_ for *M*(4) and *M*(4′) split site, respectively. Following the refinement strategies from the literature^23,25,26^, *T*(1) occupancy was fixed to Si_3.52_Al_0.48_ on the basis of EPMA chemical data, and a full Si occupancy was assumed for *T*(2) site. The *C* cations sites *M*(1), *M*(2) and *M*(3) are strongly distorted, and *M*(2) polyhedron shows a distinct relaxation, as expected in Mg-rich tremolites^26^. Site populations for tremolite asbestos, derived from EPMA based structural formula, are reported in Table 1, together with their corresponding site scattering (ss, electrons per formula unit) and mean bond length (mbl, Å)^25,26^. A satisfactory agreement can be noticed in Table 1 between this dataset and the corresponding derived from the structural refinement. A bond valence balance is reported in Supplementary Table S7, showing no significant deviation from the expected values for both cation and anions valence sums, thus confirming the soundness of the cation distribution in the refined crystal chemical model. The Raman spectrum of the tremolite sample in the region 1200–200 cm^−1^ is reported as Supplementary Information (Fig. S7) together with the relative Raman bands and the literature data for tremolite^27^ (Supplementary Table S8). Band assignments is based on^27–29^. The most intense peak at 674 cm^−1^ can be ascribed to ν_1_ symmetric stretching mode Si–O_br_–Si, and its position^28^ indicates a ^c^Mg/^c^Fe ratio X ≈ 1. This value is weakly influenced by the quite low ^*T*^Al content of the studied tremolite sample^29^. As demonstrated by the literature data, a valuable series of information on both short and long range order in amphiboles can be deduced from position, full width at half maximum (FWHM) and intensity of the bands in the OH stretching region^30–32^. This point has been made clear from a large amount of data from Fourier-transform infrared spectroscopy (FTIR), but in the last years it has been demonstrated that Raman spectra also provide the same type of information^33^. The Raman spectrum in the OH-stretching region (3500–3800 cm^−1^) is displayed in Supplementary Fig. S8. The pattern shows a rather sharp peak at 3671 cm^−1^ with a second minor peak at 3658 cm^−1^. Following the currently notation^30,34^, the former can be assigned to the MgMgMg–OH-^[A]^ and the latter to the MgMgFe–OH-^[A]^□ configurations, i.e., to local configurations associated to vacant *A*-sites. The broad band centred at 3714 cm^−1^ can be assigned to OH-dipoles close to a locally occupied the *A*-site and associated with Al at the tetrahedral sites^34^. Additional information on the cation distribution comes from the single-crystal FTIR spectrum (see Supplementary Fig. S8). The FTIR spectrum is indeed more complex than the Raman spectrum and shows, in addition to the main peak at 3672 cm^−1^, two well defined and intense absorptions at 3719 and 3690 cm^−1^. The 3719 cm^−1^ component can be assigned to local MgMgMg–OH–^[A]^Na configurations associated with Al in *T*, in line with a strong ordering in the structure between the A-cation and the Al at *T*(1)^29,33^. By considering the lack of F in the sample (Supplementary Table S2) the second component at 3690 cm^−1^ must be assigned to the same configurations but associated with Al simultaneously disordered at the octahedral C sites^35^. From decomposition of the Raman spectra into single Gaussian components the relative amounts of the divalent cation at *M*(1,3) can be calculated^35^. Accordingly, Mg_Raman_ = ^c^Mg/^c^(Mg + Fe^2+^) = 0.960, in excellent agreement with the provided by structural refinement (Mg_structural_ = ^c^Mg/^c^(Mg + Fe^2+^) = 0.960) and from EPMA (Mg_EPMA_ = ^c^Mg/^c^(Mg + Fe^2+^) = 0.972) (Table 1).

**Table 1:** Comparison between site populations (a.p.f.u), site scattering (s.s., a.p.f.u) and mean bond length (m.b.l., Å) values, derived from structural refinement and EPMA based recalculations. M.b.l. values for *T* sites in EPMA based recalculations were reported according to Oberti et al*.*^26^.

	Structural Refinement			EPMA		
Site	Population	s.s	m.b.l	m.b.l	s.s	Population
*T*(1)	Si_3.52_Al_0.48_		1.635	1.633		Si_3.53_ Al_0.48_
*T*(2)	Si_4_		1.635	1.630		Si_4_
*M*(1)	Mg_1.92_Fe_0.08_	25.12	2.078	2.073	25.12	Mg_1.92_Fe_0.08_
*M*(2)	Mg_1.92_Fe_0.08_	25.12	2.077	2.071	25.41	Mg_1.76_Al_0.14_Fe_0.02_Cr_0.05_ Ti_0.01_ Ni_0.01_Mn_0.01_
*M*(3)	Mg_0.96_Fe_0.04_	12.56	2.068	2.070	12.42	Mg_0.97_ Fe_0.03_
Σ C cat	Mg_4.80_ Fe_0.20_					Mg_4.65_ Al_0.14_Fe_0.13_Cr_0.05_ Ti_0.01_ Ni_0.01_Mn_0.01_
A cat	Na_0.29_	3.19			2.94	Na_0.25_ K_0.01_
B cat	Ca_1.92_Fe_0.08_	40.48			40.75	Ca_1.80_ Fe_0.17_ Na_0.03_

## Discussion

According to the definition of “fibre” that we have used in this work (L > 5 µm, W < 3 µm and L/W > 3)^3,13,14^, a fraction of the tremolite fibres found in the sample can be classified as tremolite asbestos. SMA1971-214 tremolite asbestos has a chemistry comparable to low-temperature amphiboles from other oceanic fracture zones (e.g., the Vema transform^10,11^, Supplementary Fig. S9). In particular, primary and secondary amphiboles from gabbroic rocks in the oceanic crust are commonly represented by pargasite and Mg-hornblende^11^. Conversely, amphiboles from oceanic peridotites are tremolite, Mg-hornblende, edenite, and pargasite following an increasing T order^11,36^. SMA1971-214 sample was collected on a peridotitic massif coherently and shows a composition in accordance to the ultramafic group. Tremolite is often recognized within mylonites and ultramylonites formed at amphibolite and green-schist facies conditions^11^. In these settings, the crystallization of secondary phases such as talc or serpentine is controlled by the occurrence of orthopyroxene as relict phase^9,37^.

**Figure 4:**
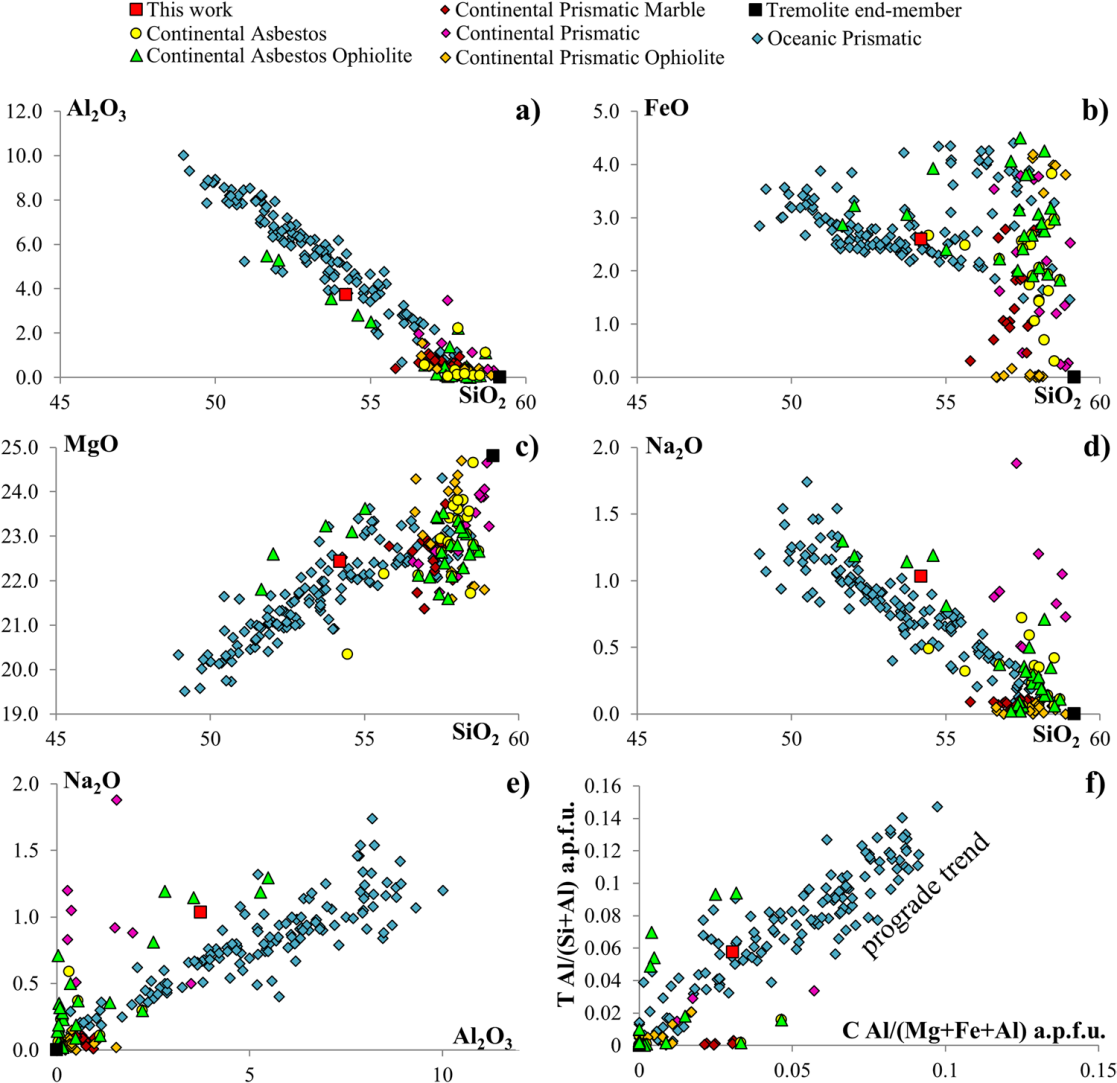
Comparison between the composition of the major elements of SMA1971-214 tremolite asbestos and that of tremolite amphiboles from oceanic and continental tectonic environments. (**a**) Al_2_O_2_ (wt%) versus SiO_2_ (wt%). (**b**) FeO (wt%) versus SiO_2_ (wt%). (**c**) MgO (wt%) versus SiO_2_ (wt%). (**d**) Na_2_O (wt%) versus SiO_2_ (wt%). (**e**) Na_2_O (wt%) versus Al_2_O_3_ (wt%). (**f**) Al/(Al + Si) in T site (a.p.f.u.) versus Al/(Al + Mg + Fe) in C site (a.p.f.u.). ± 1 σ compositional variability of tremolite SMA1971-214 is within the symbol area. See Supplementary Information for references.

Thermal constraints can be partially inferred from the SMA1971-214 paragenesis formed by tremolite + chlorite + lizardite. Thermal approximations derived from chlorite-based geothermometers^38,39^ provide T estimates of 202 ± 10 °C^38^ and 206 ± 10 °C^39^ (Supplementary Table S9). This temperature is in agreement with the occurrence of lizardite as the only serpentine phase, being lizardite more stable than chrysotile and antigorite between 200 and 320 °C^40^, having as upper limit the lizardite-antigorite boundary^5,40,41^. The association tremolite-chlorite-lizardite is stable in the zeolite/pumpellyite facies^42^ thus suggesting that the chlorite thermometric estimates may represent the minimum crystallization temperatures for tremolite. Textural relationships reveal chlorite and lizardite to form after tremolite. Hence its formation may occur in a pristine phase at higher T conditions spanning up to the green schist metamorphic facies^11^. Compositionally SMA1971-214 tremolites plot away from the pure endmember along the trend of increasing P and T (Fig. 4). Replacement of Si by Al in *T* site of Ca-amphibole is driven by temperature increasing over pressure; while the increase of Al in octahedral site is driven by P over T^43–46^ (Fig. 4f, Supplementary Fig. S9d). SMA1971-214 tremolite falls in the prograde trend for Al/(Si + Al) vs. Al/(Mg + Fe + Al) which indicates that T and P have a similar role in the crystallization process (Supplementary Fig. S9d). Constraining the upper temperature formation of tremolite is however complicated by the fact that no other high-T phase occurs in the observed paragenesis. The tremolite stability field ranges up to 700–800 °C in the CaO–MgO–Al_2_O_3_–SiO_2_–H_2_O system^41,42,47^. In natural samples, tremolite is stable below 400–650 °C between 0.1 and 0.5 GPa (greenschist to lower amphibolite facies) scaling with the CO_2_ partial pressure^40,41,47–49^. Lacking of high-T mineral reactants may suggest the formation of the observed paragenesis to occur in a fluid-dominated system at low T conditions, in agreement with the δ^18^O temperature ranging 270–350 °C estimated from tremolite-rich fault rocks from an ultramafic protolith in a similar tectonic setting at the Atlantis Massif (MAR 30° N)^50^.

As previously described, sample SMA1971-214 was collected along a major fault (Fig. 1; Supplementary Fig. S1). Rocks collected in the same site are pervasively serpentinized peridotites systematically impregnated by plagioclase^51,52^. The peculiar modal composition of these rocks can explain the lack of talc in the observed paragenesis. Talc is commonly found in association with tremolite^42,49^; its crystallization requires fluids contextually saturated in both Mg and Si^52^. A source of silica as gabbroic or basaltic rocks is hence necessary in association to the collected peridotites^52^. The complete absence of such lithologies in the studied site led us to hypothesize that the impregnating plagioclase dispersed in the host peridotite may represents the source of Ca upon hydrothermal alteration while the Si content, dominated by the bulk ultramafic host, remains undersaturated.

Alternatively, Ca can be supplied by leaching of carbonate phases. However, the incorporation of carbonate within the migrating fluids also enriches them in CO_2_ pointing to re-precipitation of new carbonates along with tremolite in Si-undersaturated environment^53^. Carbonate phases are absent in our sample thus confirming plagioclase as the only possible source of Ca in the system. Overall the tremolite-chlorite-lizardite paragenesis appear to be formed in a shear-driven, fluid-dominated environment across the lower green-schist to the zeolite-pumpellyite facies condition^11,42^.

In the following we attempt a first order comparison of our results with occurrences from the continental settings. The general lack of detailed crystallographic descriptions in literature hinders the possibility of strict correlations, however, continental tremolites commonly show higher SiO_2_, MnO, MgO and CaO and lower TiO_2_, Al_2_O_3_ and Na_2_O than oceanic occurrences and are in general closer to the tremolite end-member composition (Fig. 4). FeO in oceanic tremolite is always > 1.4 wt% while in continental tremolite often decreases below the detection limit (Fig. 4b). Na_2_O and Al_2_O_3_ are on average very low or absent in continental tremolites while forming wide negative trends with Si in oceanic ones (Fig. 4a,d). This character, associated with the dispersion in Si and Na suggests a compositional drift toward the pargasitic end-member. Overall continental and oceanic tremolites plot on different trends clearly visible in the Fe–Si and Mg–Si relationships (Fig. 4b,c). These trends are followed by both prismatic and fibrous (asbestos) tremolite in both tectonic settings. Being in general, the continental tremolite sampled on ophiolitic massifs, this observation points to their formation under conditions different from the primary oceanic settings, hence possibly formed during obduction or the orogenetic cycles postdating the tectonomagmatic building of the former oceanic crust.

It is also worth noting that tremolites grown along faults at low T/P conditions, as SMA1971-214, in both continental and oceanic settings plot on a trend slightly diverging from that of the oceanic ones. These differences could be ascribed to different formation processes. Our sample is similar in composition to asbestos specimens growth along/near fault and fractures systems in ophiolites of southern Italy (Pollino Massif)^54^. The data from continental asbestos tremolites show a wider compositional range with the previous literature^55^. Dorling and Zussman^55^ show that asbestos tremolite is compositionally closer to tremolite end-member with respect to prismatic ones and suggest that increase of Na in the *A* site is compensated by substitution of Ca by Na in *M*(4) site. Our dataset instead shows a direct correlation between Na_2_O and Al_2_O_3_ thus suggesting that the increase of Na content is at least partially compensate by Al in *T* sites (Fig. 4). The differences in terms of Na content between our sample and continental asbestos can be related to the occurrence of feldspar^51^ alteration by the SMA1971-214 migrating fluids and by contribution from sea water. As a matter of fact, the incorporation of Cl in tremolite is inhibited by the high Mg content^56^ (Supplementary Table S2) and thus the sea water imprinting is poorly constrained.

We now compare the structural features of our sample with the published data of prismatic or fibrous natural tremolites of different origin (Table 2): (1) the prismatic sample from Gouverneur Mining District (NY, USA) where metamorphism at 500–600 °C between quartzite and dolomite occurred and followed later by serpentinization^57,58^, (2) the fibrous sample (tremolite asbestos) from Susa Valley (Piedmont region, Italy) where serpentine/chlorite schists of the “Unità Oceanica della bassa Val di Susa (zona piemontese)” occur in tectonic contacts and fault zones^59^, (3) the fibrous sample (tremolite asbestos) from Ala di Stura (Lanzo Valley, Piedmont region, Italy) where serpentinites of the ultrabasic Lanzo massif in the western Alps are part of the sub-continental lithosphere emplaced at shallow levels during the opening of the Mesozoic Piemontese-Ligurian basin^60^ (Table 2). The lattice constants *a*, *c* and *β* are comparable among the samples whereas the *b* value from the Gouverneur Mining District is shorter, yielding a smaller calculated volume (905.733(3) Å^3^). Our sample has the highest concentration of Al in the *T*(1) site (0.48 a.f.u.). The two samples from the Italian Alps (Susa Valley and Ala di Stura) have no Al in the *T*(1) site. None of the samples display Al or other metals in the *T*(2) site. These differences in terms of Al content could be ascribed to the distinct composition of protolith and different T-P conditions. As mentioned above, the substitution of Si with Al increases with T that promotes the exchange in the *T* site, while P favours the exchange in the octahedral *M* site^43–45^. Tremolite from the Alps ophiolite developed mainly in tremolite + calcite veins formed at last stages of ophiolite emplacement almost at surficial conditions by CO_2_-rich fluids^61^. Consequently, Alps tremolite formed under lower T-P conditions than SMA1971-214 (Fig. 4c). In addition, peridotites collected nearby sample SMA1971-214 showed different degrees of feldspar impregnation^51,62^ pointing to a higher Al, Na and K content in the oceanic protolith being the Alps one formed within lherzolite mantle rocks^61^. Concerning tremolite from Gouverneur Mining District, the low Al content in the crystal structure reflects the primary protolith composition (quartzite and dolomite)^63^. The concentration of Mg at the *M*(1,2,3) sites is comparable (Table 2). Mn and Fe deficiency in SMA1971-214 tremolite asbestos can be related to the fluid composition and preferential partitioning into the chlorite phase that shows an order of magnitude higher concentration of both oxides (Supplementary Table S7). The B site (i.e., *M*(4)) shows comparable Ca contents, minor Fe (0.08) in our sample and minor Na and K contents in the other three samples (Table 2). The occupancy at the *A* site is different in the examined samples: SMA1971-214 tremolite asbestos contains 0.29 a.f.u. of Na, the sample from Gouverneur Mining District contains both K (0.12) and Na (0.18) while the *A* sites are empty in both samples from the Italian Alps (Table 2).

**Table 2:** Site populations and unit cell parameters of tremolite SMA1971-214 compared to reference data: (1) tremolite from Gouverneur Mining District (NY, USA)^57,58^. (2) tremolite from Susa Valley (Piedmont region, Italy)^59^. (3) tremolite from Ala di Stura (Lanzo Valley, Piedmont region, Italy)^60^.

Parameter	Tremolite SMA1971-214	(1)	(2)	(3)
*T*(1)	Si_3.52_Al_0.48_	Si_3.80_Al_0.20_	Si_4_	Si_4_
*T*(2)	Si_4_	Si_4_	Si_4_	Si_4_
*M*(1)	Mg_1.92_Fe_0.08_	Mg_2_	Al_0.01_Fe^2+^_0.05_Mg_1.92_Mn_0.02_	Mg_1.88_Fe^2+^_0.09_Mn_0.03_
*M*(2)	Mg_1.92_Fe_0.08_	Mg_1.94_Fe_0.06_	Fe^2+^_0.04_Fe^3+^_0.02_Mg_1.94_	Mg_1.86_Fe^2+^_0.12_Fe^3+^_0.03_
*M*(3)	Mg_0.96_Fe_0.04_	Mg	Fe^2+^_0.02_Mg_0.98_	Mg_0.95_Fe^2+^_0.05_
A	Na_0.29_	K_0.12_Na_0.18_	–	–
B	Ca_1.92_Fe_0.08_	Ca_1.8_Na_0.2_	Ca_1.95_Na_0.05_K_0.01_	Ca_1.96_Na_0.01_K_0.01_
*a*	9.8469(6) Å	9.85145(1) Å	9.84174(8) Å	9.8424(1) Å
*b*	18.0651(11) Å	18.02911(2) Å	18.05932(19) Å	18.0715(2) Å
*c*	5.2795(4) Å	5.273416(5) Å	5.27856(6) Å	5.28354(7) Å
V	907.98(9) Å^3^	905.733(2) Å^3^	907.37(1) Å^3^	909.07(2) Å^3^
*β*	104.803(3)°	104.7566 (1)°	104.732 (1)°	104.68(1)°

Although minor differences in terms of morphological and crystal structural features were found, our study shows that the tremolite asbestos from the MAR plot on different compositional trends with respect to both tremolite asbestos and prismatic tremolite from the continental setting. Primary, oceanic, formation conditions can be occasionally preserved in fault associated paragenesis on obducted ophiolitic units.

Taken together, the data presented in this paper may provide valuable information for future studies focusing on the differences between fibrous amphiboles (amphibole asbestos) from the oceanic and continental environment.

## Materials and methods

### Geological setting

The Romanche Transform Fault (RTF) is the largest transform in the Equatorial Mid Atlantic Ridge. The fracture zone crosses the Atlantic Ocean from South America to West Africa for approximately 3200 km^64^. The transform fault domain offsets the MAR by ca. 950 km^64^^.^ The RTF is a complex mega-transform formed by a main deep E–W-trending valley flanked by steep ridges and by a secondary system of oblique troughs and ridges^64^. Between 18° and 19° W longitude the transform valley reaches the deepest point of the Atlantic ridge at 7.8 km below the sea level. The southern MAR segment meets the transform between 17° and 16° W^51^. This Ridge-Transform Intersection has been recently explored by the SMARTIES expedition in 2019 on board the Research vessel “PourquoiPas?”^15^. The multibeam bathymetric survey revealed complex tectonics dominated by a 20 km large detachment forming a large core complex on the eastern flank of the MAR ridge. The main detachment surface is dissected by a set of west-dipping normal faults (Fig. 1). Sample SMA1971-214 was collected on the exposed plane of one of these faults during the 1971 dive of the manned submarine Nautile^15^ (Supplementary Fig. S1). Bathymetry and 3D rendering (Fig. 1) have been generated from shipborne multibeam echosounding data acquired during the SMARTIES cruise^15^. Data were acquired with a Reson Seabat 7150 echosounder, using 880 beams at 12/24 kHz with an average swath of 12 km. Data were cleaned onboard and processed for 2 and 3D rendering with the Globe software by IFREMER^65^.

### Electron and optical microscopy

Morphological observations were performed using a Field Emission Gun (FEG) Scanning Electron Microscope (SEM) FEI Nova NanoSEM 450 FEG-SEM equipped with an Energy Dispersive X-ray (EDX) spectrometer at the Centro Grandi Strumenti of the University of Modena and Reggio Emilia (CIGS-UNIMORE). Operating conditions were 15 kV accelerating voltage and 3.5 μA emission current, 20 nA beam current and 6 mm working distance. An aliquot of raw sample was suspended in distilled water and fixed on an aluminium stub with double-stick carbon tape and then sputter-coated with gold (10 nm of thickness), using a gold sputter coater Emitech K550. Images were acquired using the signal of secondary electrons. EDX spectra were constantly collected to confirm the chemistry of the observed minerals^66^.

Transmission electron microscope (TEM) investigations were carried out at CIGS-UNIMORE by using a Talos F200S G2 microscope, equipped with S-FEG Schottky field emitter operating at 200 kV and two large‐area EDX spectrometers with Silicon Drift Detectors (SDD). A small amount of sample was suspended with 1 mL of ethanol in a test tube, sonicated for 1 min (using a low power sonic bath) and left to set for 5 min. A drop of the suspension was then transferred and dried onto a 300-mesh carbon copper TEM grid.

Polarized Light Optical Microscopy (PLOM) investigation was performed in transmitted light through an Olympus CHA on crystals embedded in epoxy resin and subsequently polished to a thickness of 30 µm.

### Thermal analysis coupled with evolved gases mass spectrometry

Thermogravimetric and differential thermal analysis (TGA and DTA) measurements were performed at the Department of Chemical and Geological Science, University of Modena, with a Seiko SSC 5200 thermal analyser coupled with a quadrupole mass spectrometer ESS, GeneSys Quadstar 422 to identify the gases evolved during heating (i.e., mass spectrometry of evolved gas analysis, MS-EGA) like described in detail in Kamruddin et al.^67^ and Malferrari et al.^68^ Gas sampling by the spectrometer was via an inert, fused silicon capillary system, heated to prevent the condensation of gases. Measurements were performed on air-dried samples under the following experimental conditions: heating rate: 20 °C/min; heating range: 20–1200 °C; TG and DTA data measurement: every 0.5 s; purging gas: ultrapure helium, flow rate: 100 μL/min. MS-EGA were carried out in multiple ion detection (MID) mode measuring the signal of the m/z ratios 17 and 18 for H_2_O, 28 and 44 for CO_2_, 30 for NO, 34 for H_2_S, 46 for NO_2_ and 64 for SO_2_ (m/z is the dimensionless ratio between the mass number (m) and the charge (z) of an ion); secondary electron multiplier detector at 900 V were employed with 1 s integration time on each measured mass. To avoid differences in relative humidity, samples were isothermally equilibrated at 25 °C for 15 min inside the oven using a 100 μL/min flow of ultrapure He.

### Quantitative chemical analysis

Quantitative chemical composition of the carbon-coated sample was obtained at the Department of Earth Sciences, University of Milan, using a JEOL 8200 SuperProbe Electron Probe Microanalyzer equipped with a Wavelength-Dispersive X-Ray (WDS) spectrometer system, W hairpin type filament. Detectable wavelength is 0.087 to 9.3 nm. Atomic number resolution on BSE (Z): less/equal than 0.1 (CuZ). The following analytical conditions were used: excitation voltage of 15 kV, specimen current of 5 nA, peak-count time of 30 s, background-count time of 10 s. The instrument is also equipped with EDX system characterized by a detectable element range: Na to U, energy resolution: 144 eV and lithium (Li)-doped silicon single-crystal semiconductor detector. The following elements were mesured at each analytical spot: Si, Ti, Al, Cr, Mn, Mg, Ca, Na, K, Ni, Fe, Pb, Co, V, Cu, F and Cl. Calibration used a set of standards: elemental vanadium for V, elemental chromium for Cr, elemental cobalt for Co, elemental copper for Cu, nickeline for Ni, galena for Pb, omphacite for Na, orthoclase for K, rhodonite for Mn, forsterite for Mg, fayalite for Fe, ilmenite for Ti, grossular garnet for Al, Si and Ca, fluorite for F and scapolite for Cl. F and Cl were below detection limit in all analysed points. The raw data were corrected for matrix effects using the Phi-Rho-Z method from the JEOL series of programs.

### Mössbauer spectroscopy

Room Temperature Mössbauer spectra were collected at the Department of Chemical Science (University of Padova) by means of a conventional constant acceleration spectrometer mounting an ^57^Co source (nominal strength 1850 MBq). To avoid, or minimize, textural effects, the absorber was prepared by mixing ≈ 70 mg of sample, gently crushed in acetone, with vaseline. The amount of sample was estimated in order to respect the thin absorber thickness limit^69^. The spectrum was fitted with a Voigt-based fitting procedure (VBF) providing a distribution of quadrupole splitting distributions (QSD) by using Recoil software^70^, the reduced χ^2^ method was used to evaluate the goodness of the fitting procedure. The centre shift (δ_0_) is quoted relative to α-Fe foil.

### Single-crystal diffraction data collection, refinement and structure analysis

Single-crystal X-ray diffraction data were collected at Earth Science Department of Pisa University, using a Bruker Smart Breeze diffractometer with an air-cooled CCD detector, with graphite-monochromatised Mo Ka radiation. Relevant details for single crystal data collection are reported as supplementary information.

### Vibrational spectroscopy (FTIR, Raman)

Single-crystal FTIR spectra in the OH-stretching medium-infrared (MIR) region were collected with unpolarized light using a Bruker Hyperion 3000 microscope equipped with an MCT (Mercury Cadmium Telluride) detector and a KBr beam splitter at Istituto Nazionale di Fisica Nucleare (INFN, Frascati, Rome). The unpolarized micro-Raman spectrum for tremolite was obtained on a polished sample employed for EPMA investigations, working in backscattered geometry with a Jobin–Yvon Horiba XploRA Plus apparatus, equipped with a motorized x–y stage and an Olympus BX41 microscope with a 100·/0.75 objective lens. The Raman spectra were excited by the 532 nm emission of a solid-state laser attenuated to 25% intensity, and the system was calibrated using the 520.5 cm^−1^ Raman band of Si before every experimental session. Spectra were collected through repeated multiple acquisitions with single counting times of 30 s, and backscattered radiation were analysed with a 1200 mm^−1^ grating monochromator.

## Supplementary Information

Supplementary Information 1.

Supplementary Information 2.
