# Supplementary material for: Occurrence and characterization of tremolite asbestos from the Mid Atlantic Ridge
Source: Sci Rep. 2021 Mar 18;11:6285. doi: 10.1038/s41598-021-85576-w (PMC7973559; doi:10.1038/s41598-021-85576-w)
Supplement: Supplementary file 1 — Supplementary Information 1. [file 41598_2021_85576_MOESM1_ESM.pdf]

SUPPLEMENTARY INFORMATION

Occurrence and characterization of tremolite asbestos from the Mid Atlantic Ridge

Dario Di Giuseppe[1,2*], Natale Perchiazzi[3], Daniele Brunelli[1,4*], Tommaso Giovanardi[1], Luca Nodari[5], Giancarlo Della Ventura[6,7], Daniele Malferrari[1], Marcia Maia[8], & Alessandro F. Gualtieri[1]

[1]Department of Chemical and Geological Sciences. University of Modena and Reggio Emilia, Modena, Italy.
[2]Department of Sciences and Methods for Engineering. University of Modena and Reggio Emilia, Emilia, Italy.
[3]Department of Earth Sciences. University of Pisa, Via S. Maria 53, I-56126 Pisa, Italy.
[4]CNR-ISMAR Institute for Marine Sciences, Italian National Research Council, Via Gobetti 10, 41100 Bologna, Italy.
[5]CNR-ICMATE Institute of Condensed Matter Chemistry and Technologies for Energy. Italian National Research Council, Corso Stati Uniti, 4, I-35127, Padova, Italy.
[6]Department of Sciences. University of Roma Tre, Roma, Italy.
[7]INFN Laboratori Nazionali di Frascati, Frascati (Roma), Italy.
[8]CNRS- Géosciences Océan UMR 6538 - Institut Universitaire Européen de la Mer, Plouzané, France

*email: dario.digiuseppe@unimore.it – daniele.brunelli@unimore.it

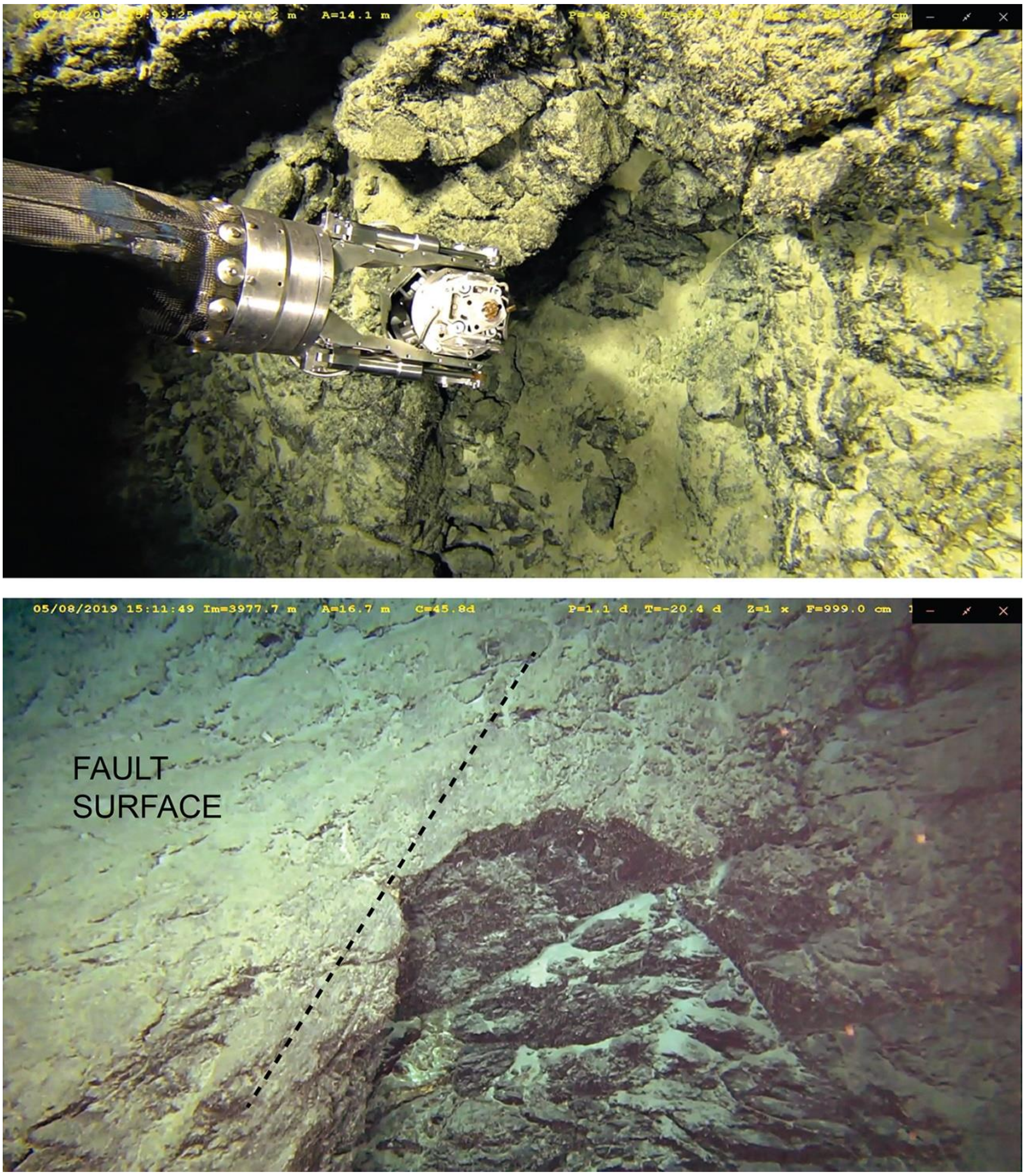

Figure S1. Frames of the video taken during the rock sampling activity of the Nautile submarine. The images show the moment when the sample SMA1971-214 was collected.

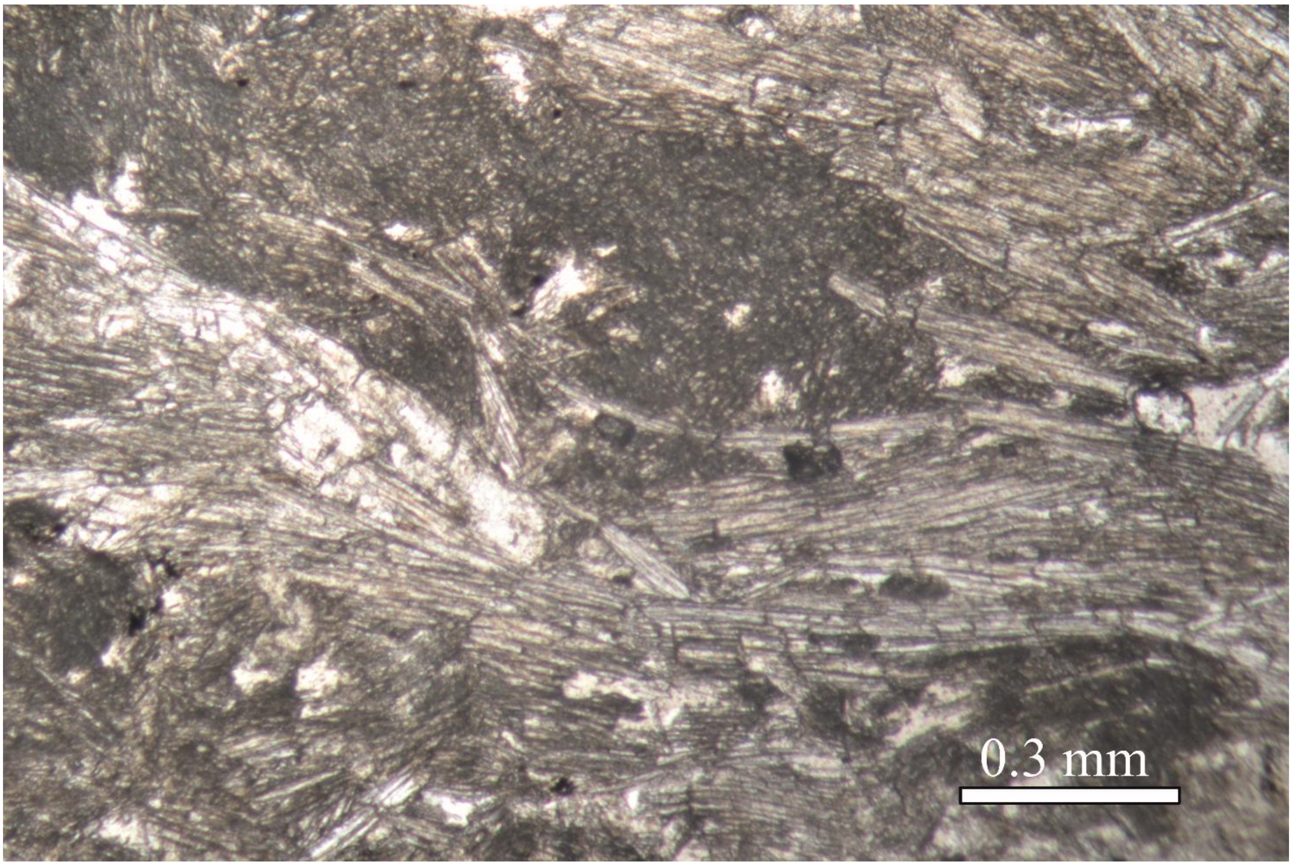

Figure S2. Sample SMA1971-214 viewed under polarized light optical microscopy, (plane-polarized light). Tremolite fibres display a wavy orientation, with late kink cleavage and are commonly folded, suggesting deformation processes subsequent to crystallization. Fibres show the typical sigma-like structure.

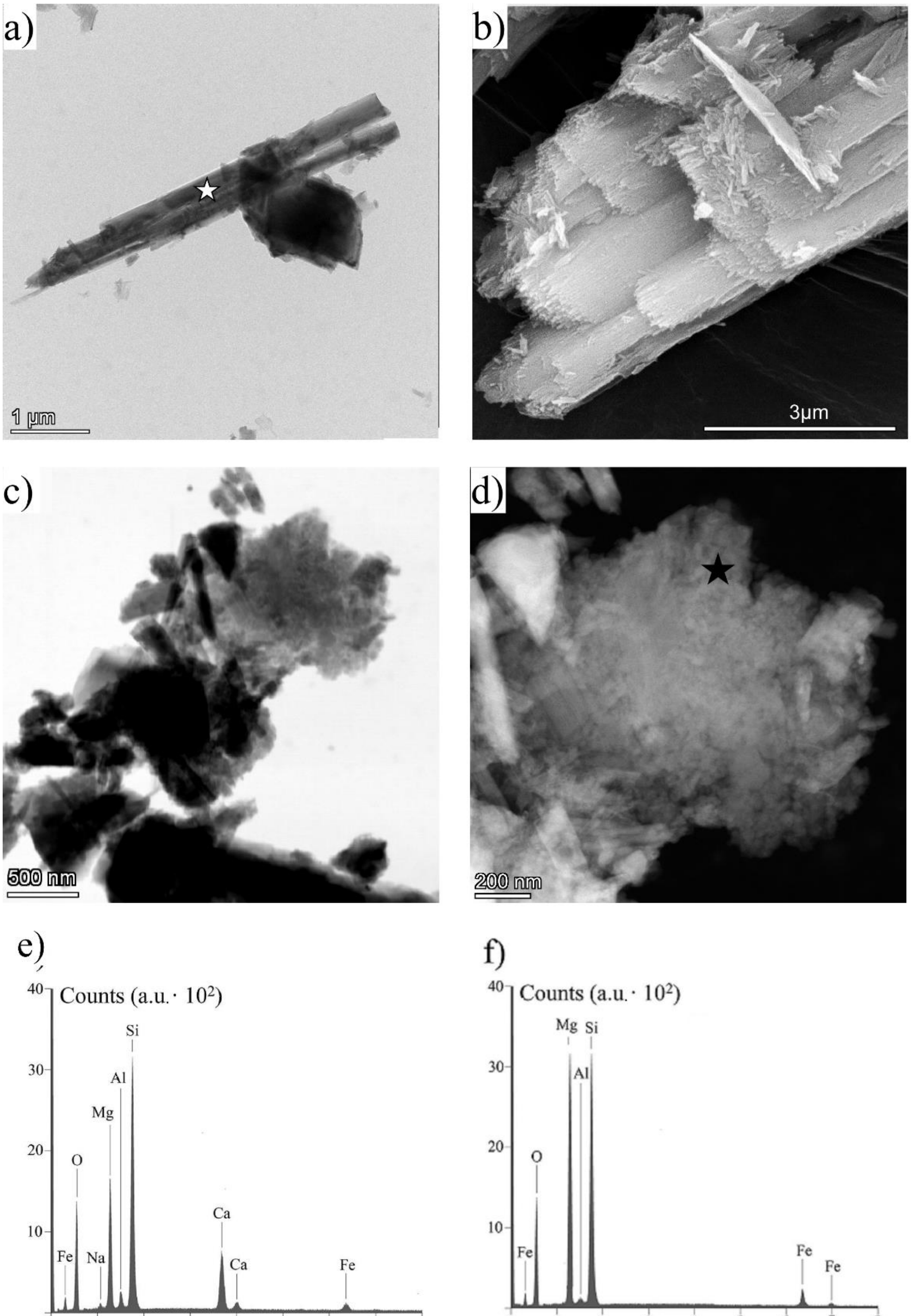

Figure S3. Representative TEM and SEM images of the examined sample. **a**) TEM image of tremolite fibres. The white stars show the EDX analysis spots. **b**) SEM image of a tremolite fibres bundles as viewed perpendicular to the long axis of the fibres. **c**) Prismatic tremolite and lizardite aggregates. **d**) Close up of the lizardite cluster shown in (c); the image shows lizardite particles characterized by a non-fibrous habit. The black stars show the EDX analysis spots. **e**) TEM-EDX spectrum of the tremolite shown in (a). **f**) TEM-EDX spectrum of the lizardite shown in (d).

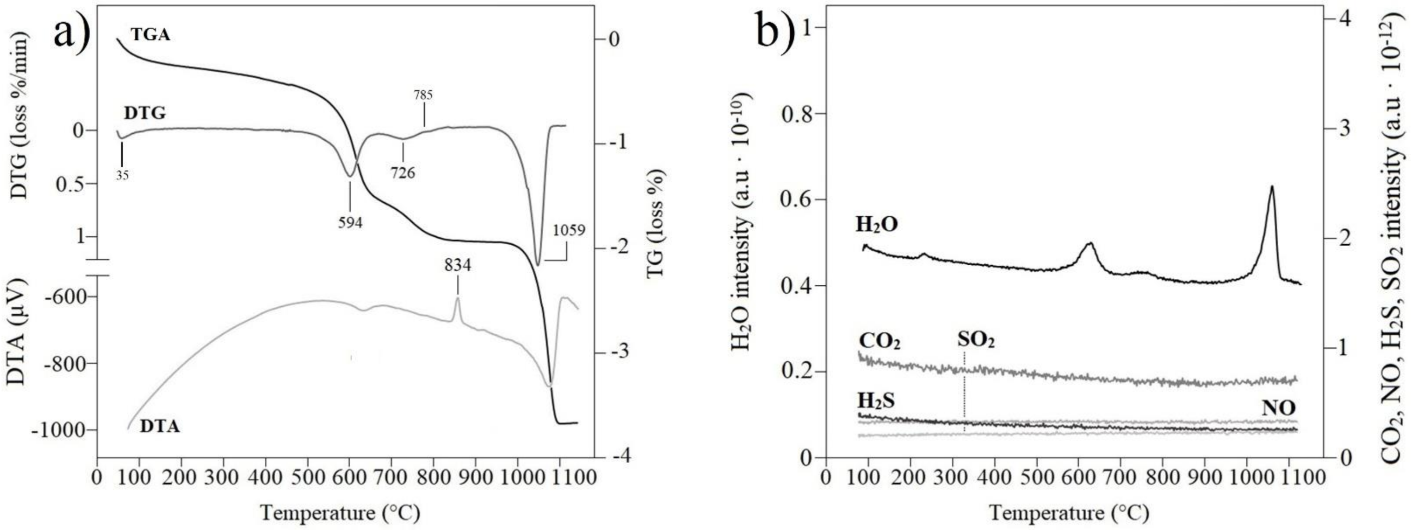

Figure S4. Thermal analysis and evolved gases mass spectrometry. **a**) TGA, DTG and DTA. **b**) MS-EGA.

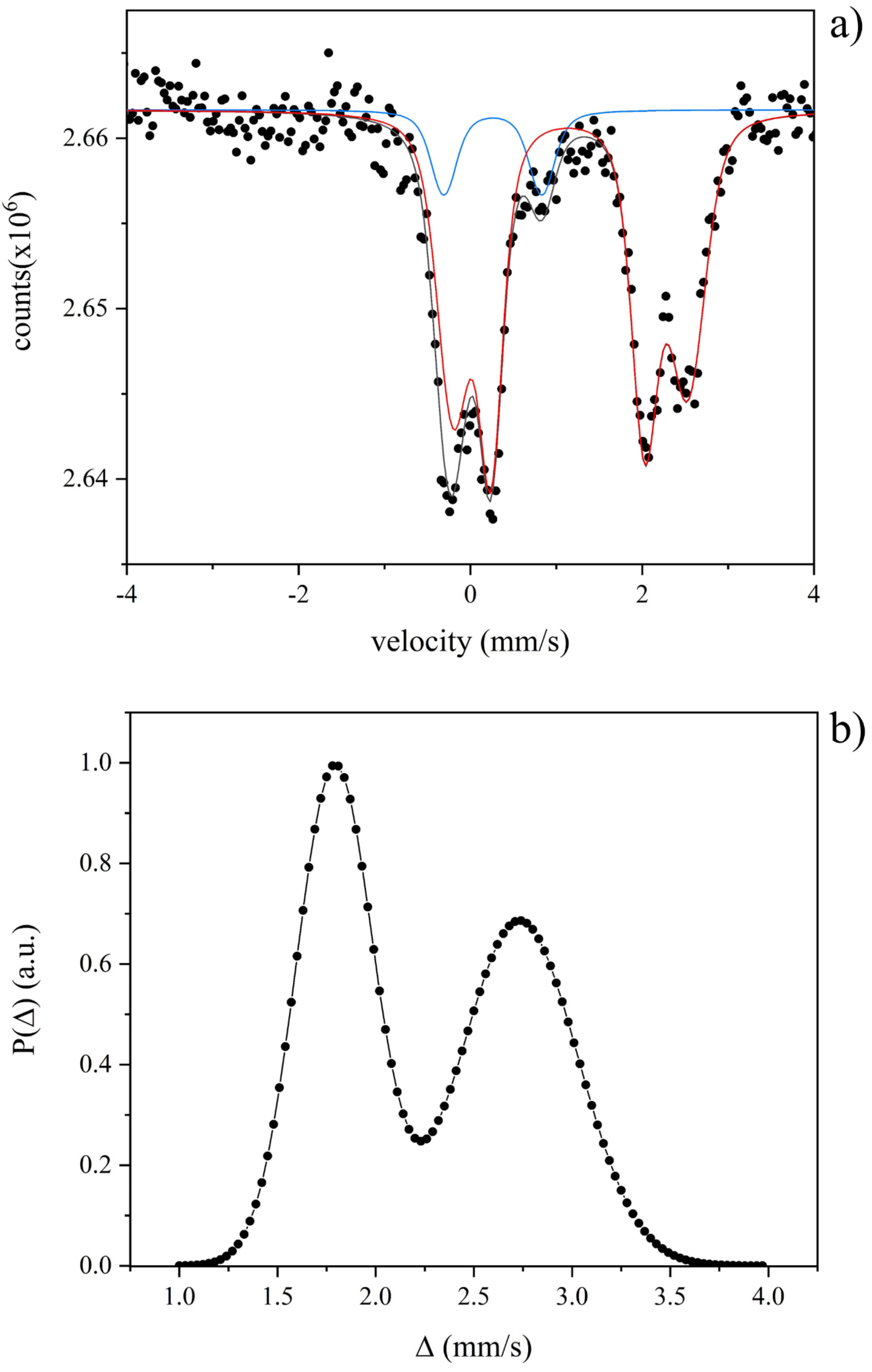

Figure S5. a) Room Temperature Mössbauer spectrum of tremolite: blue line stands for Site 1, red line stands for Site 2. **b**) QSD distribution for ferrous site.

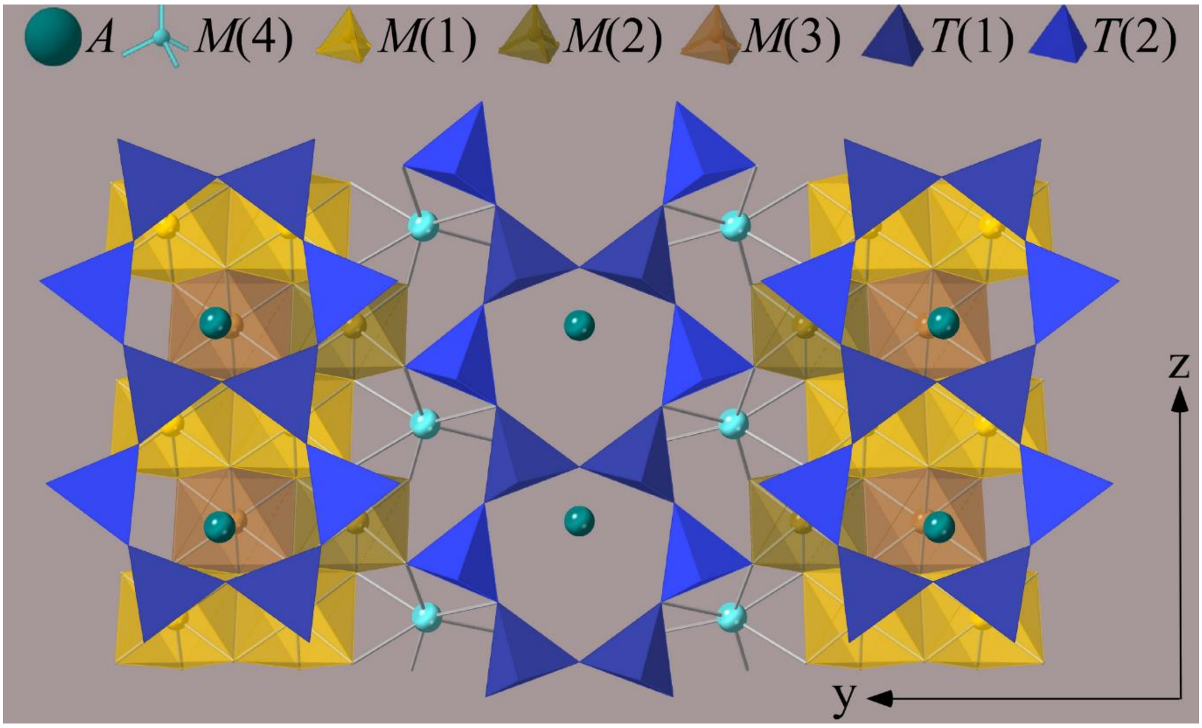

Figure S6. Crystal structure of tremolite asbestos of the SMA1971-214 sample projected onto (100).

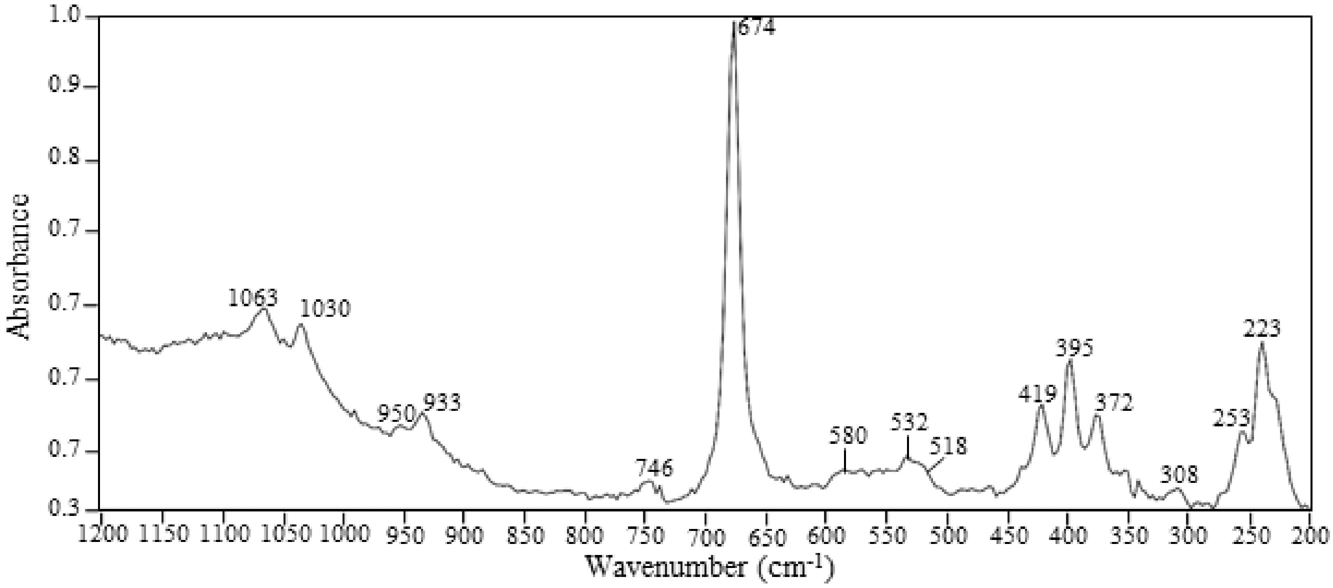

Figure S7. Raman spectrum of the sample in the 200-1200 cm^{-1} spectral range.

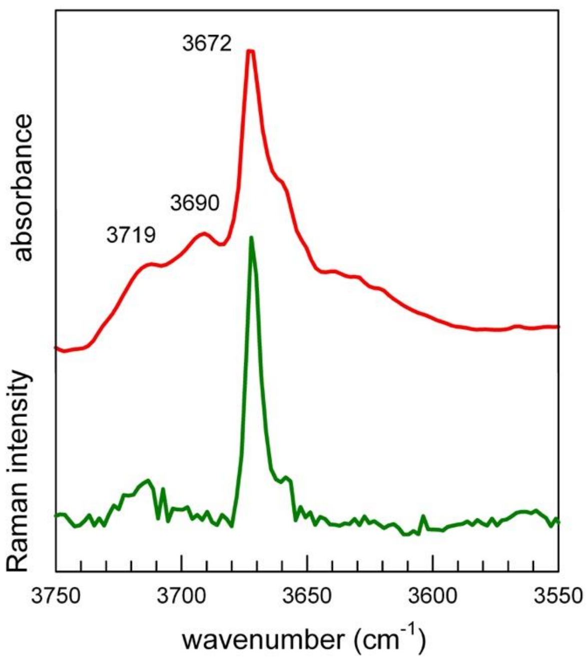

Figure S8. Raman spectrum of the studied tremolite (below) compared with the single-crystal FTIR spectrum (in red).

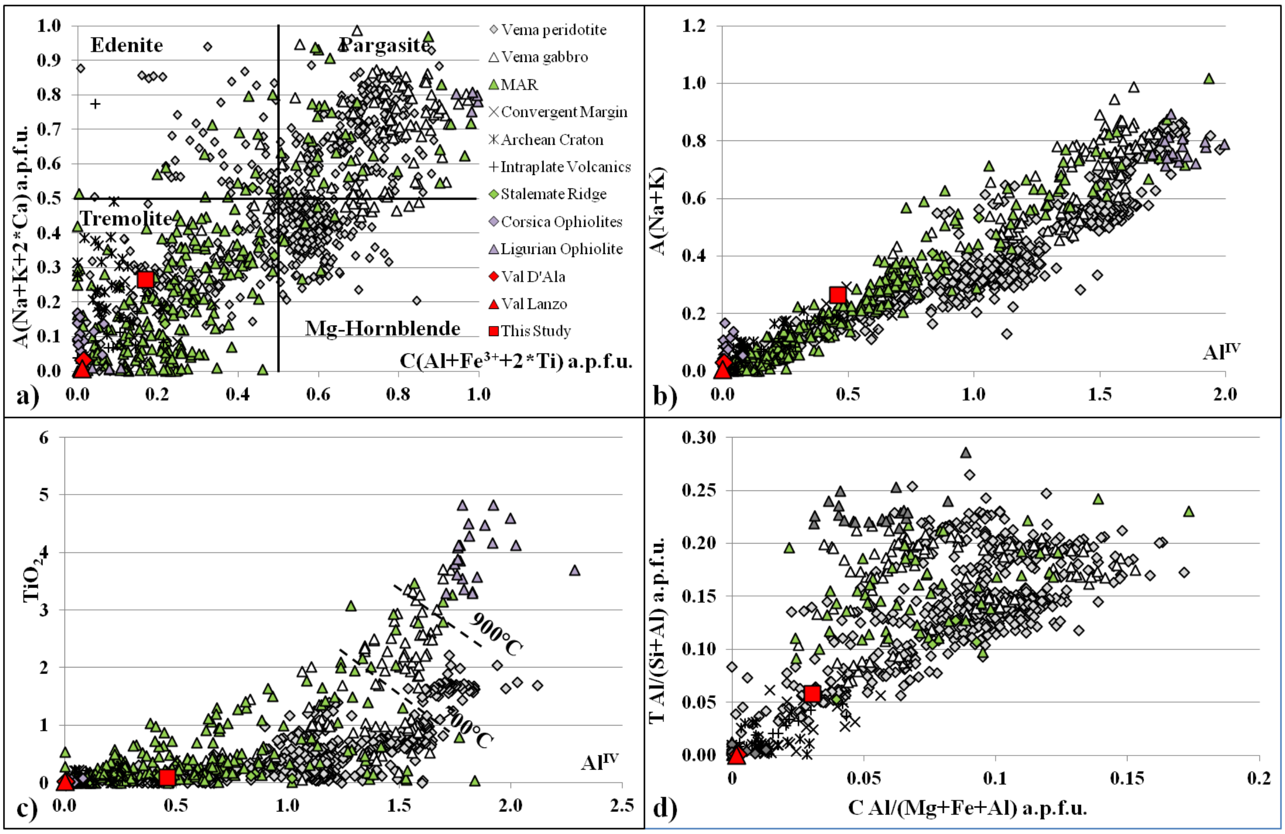

Figure S9. a) Major element composition of SMA1971-214 tremolite asbestos compared to amphiboles found in mafic and ultramafic rocks from different geodynamic contexts. Amphiboles from Vema gabbro and peridotite are from Brunelli *et al.*[1] and Cipriani *et al.*[2], respectively. Others are from GEOROC[3] and EarthChem[4]. Calcium amphibole nomenclature according to Hawthorne *et al.*[5]. Cations are calculated based on 23 oxygens. **b)** Na+K in A site (a.p.f.u.) vs Al^{IV} (a.p.f.u.). **c)** TiO_2 (wt.%) vs Al^{IV} (a.p.f.u.); isotherms are from Ernst and Lui[6]. **d)** Al/(Al+Si) in T site (a.p.f.u.) vs Al/(Al+Mg+Fe) in C site (a.p.f.u.). ± 1 σ compositional variability of tremolite SMA1971-214 is within the symbol area (see also Table S2 for standard deviation values).

		Percentiles							
	Min	5th	25th	50th	75th	95th	Max	σ	$\bar{x}$
L (μm)	5.12	7.33	10.6	16.1	22.1	73.0	93.4	19.6	22.3
W (μm)	0.17	0.29	0.54	1.05	1.57	2.68	3.03	0.72	1.17
L/D	4.76	5.68	10.7	17.0	30.0	75.4	96.3	22.7	24.6

Table S1. Summary statistics of the geometry of tremolite fibres. L (length); W (width); Min (minimum); Max (maximum); σ (standard deviation); $\bar{x}$ (average).

	Wt%	σ
SiO_2	54.21	0.58
TiO_2	0.09	0.03
Al_2O_3	3.73	0.32
Cr_2O_3	0.44	0.20
MnO	0.07	0.03
MgO	22.44	0.41
CaO	12.10	0.32
Na_2O	1.03	0.11
K_2O	0.02	0.01
NiO	0.10	0.03
FeO_{tot}	2.59	0.21
PbO	0.03	0.01
CoO	0.03	0.02
V_2O_5	0.04	0.02
CuO	0.06	0.04
F	bdl	-
Cl	bdl	-
Total:	96.98	0.36

Table S2. Average chemical composition of samples from EMPA analysis. Reported chemical compositions are mean values of several analyses carried out on 33 EMPA spot analyses. Wt% (weight percent); σ (standard deviation); bdl (below detection limit).

	δ_0 (mm/s)	δ_1 (mm/s)	$<\Delta>$(mm/s)	σ_Δ (mm/s)	p (%)	A (%)
**Site 1**	0.26±0.07		1.13±0.09	0.2±0.08	100	12±3
**Site 2**	1.07±0.05	0.03±0.02	2.73±0.05	0.29±0.06	52	88±3
			1.79±0.03	0.19±0.04	48	

Table S3. Hyperfine parameters calculated from the tremolite spectrum, χ^2: 0.65, d0, center shift, δ_1, coupling parameters between the center shift and the quadrupole splitting, $<\Delta>$ the average quadurpole splitting of the component, σ_Δ the Gaussian width of the component, p the weight of the component, A relative area.

Identification code	ShelxL
Wavelength	0.71073 Å
Crystal system	Monoclinic
Space group	*C*2/*m*
Unit cell dimensions	*a* = 9.8469(6) Å
	b = 18.0651(11) Å
	c = 5.2795(4) Å
	β= 104.803(3)°
Volume	907.98(9) $Å^3$
Z	2
Crystal size	0.01 x 0.03 x 0.2 mm
Θ range for data collection	3.992-29.99°.
Index ranges	-13<=h<=13. -25<=k<=24. -7<=l<=7
Reflections collected	11055
Independent reflections	1276 [R(int) = 0.0355]
Completeness to Θ = 25.24°	99.1 %
Absorption correction	SADABS multiscan
Max. and min. transmission	0.7469-0.6349
Refinement method	Full-matrix least-squares on F^2
Data / restraints / parameters	1276 / 0 / 108
Goodness-of-fit on F^2	0.694
Final R_1 indices obs. all data	0.030. 0.032
Largest diff. peak and hole	1.213 and -0.916 $e^-.Å^{-3}$

Table S4. Crystal data and structure refinement for SMA1971-214 tremolite asbestos.

Site	Occupancy	x	y	z	U_{eq}
A	$Na_{0.29}$	0	½	0	0.128(14)
M(1)	$Mg_{0.96}Fe_{0.04}$	0	0.08813(6)	½	0.00674 (39)
M(2)	$Mg_{0.96}Fe_{0.04}$	0	0.17645(6)	0	0.00652 (39)
M(3)	$Mg_{0.96}Fe_{0.04}$	0	0.0	0	0.00602 (55)
M(4)	$Ca_{0.96}$	0	0.27780(11)	½	0.01041(28)
M(4')	$Fe_{0.04}$	0	0.2519(27)	½	0.030(8)
T(1)	$Si_{0.88}Al_{0.12}$	0.28048(7)	0.08425(4)	0.29753(12)	0.00623(16)
T(2)	Si 1	0.28922(7)	0.17143(4)	-0.19456(12)	0.00653(16)
O(1)	O 1	0.11042(18)	0.08624(10)	0.21774(32)	0.00860(33)
O(2)	O 1	0.11910(17)	0.17168(10)	0.72508(32)	0.00842(33)
O(3)	O 1	0.10952(27)	0	0.71537(48)	0.01040(47)
O(4)	O 1	0.13385(19)	0.25176(10)	0.20990(34)	0.01115(34)
O(5)	O 1	0.34730(18)	0.13575(10)	0.10187(33)	0.01119(35)
O(6)	O 1	0.34404(18)	0.11736(10)	0.59423(33)	0.01094(35)
O(7)	O 1	0.33803(27)	0	0.28505(52)	0.01217(49)
H	H 1	0.19091	0.01300	0.76970	0.140(27)

Table S5. Fractional atomic, refined occupancies and displacement coordinates for SMA1971-214 tremolite asbestos.

T(1) $Si_{0.88}$ $Al_{0.12}$		*T*(2) Si_{1}	
O(1)	1.619(2)	O(4)	1.593(2)
O(7)	1.631(1)	O(2)	1.619(2)
O(6)	1.644(2)	O(5)	1.654(2)
O(5)	1.647(2)	O(6)	1.672(2)
<*T*1-O>	1.635	<*T*2-O>	1.635
QE	1.0012	QE	1.0048
AV	5.28	AV	19.43
M(1) $Mg_{0.96}Fe_{0.04}$		*M*(2) $Mg_{0.96}Fe_{0.04}$	
O(1) x2	2.057(2)	O(4) x2	2.017(2)
O(2) x2	2.087(2)	O(2) x2	2.088(2)
O(3) x3	2.089(2)	O(1) x2	2.126(2)
<*M*(1)-O>	2.078	<*M*(2)-O>	2.077
QE	1.0112	QE	1.0071
AV	36.64	AV	22.56
M(3) $Mg_{0.96}Fe_{0.04}$		*M*(4) $Ca_{0.96}$	
O(3) x2	2.061(2)	O(4) x2	2.310(2)
O(1) x4	2.072(2)	O(2) x2	2.398(2)
		O(6) x2	2.566(2)
		O(5) x2	2.739(2)
<*M*(3)-O>	2.068	<*M*(4)-O>	2.503
QE	1.0147		
AV	47.70		
A $Na_{0.29}$		*M*(4') $Fe_{0.04}$	
O(7) x2	2.457(3)	O(2) x2	2.04(3)
O(5) x4	2.996(2)	O(4) x2	2.262(2)
O(6) x4	3.124(2)	O(6) x2	2.93(4)
<*A*-O>	2.939	<*M*(4')-O>	2.411
T(1)	O(5)	*T*(2)	135.8(1)
T(1)	O(6)	*T*(2)	137.6(1)
T(1)	O(7)	*T*(1)	137.8(3)
O(5)	O(6)	O(5)	165.6(1)
O(6)	O(7)	O(6)	105.3(1)

Table S6. Refined occupancies, selected bond distances (Å) and angles (°) for coordination polyhedral in SMA1971-214 tremolite asbestos. Quadratic elongation (QE) and bond angle variance (AV), are reported following by Robinson et al.[7].

	T(1)	*T*(2)	*M*(1)	*M*(2)	*M*(3)	*M*(4)	*A*	*H*	∑	∑(H)
O(1)	1.022		0.752^{x2}	0.638^{x2}	1.444 ^{x4}				2.08	
O(2)		1.012	0.692^{x2}	0.686 ^{x2}		0.596 ^{x2}			2.00	
O(3)			0.688^{x2}		0.742 ^{x2}			0.81	1.06	1.86
O(4)		1.088		0.810^{x2}		0.756 ^{x2}			1.87	
O(5)	0.948	0.923				0.238 ^{x2}	0.039 ^{x2}		2.01	
O(6)	0.957	0.877				0.378 ^{x2}	0.039 ^{x2}		2.04	
O(7)	0.990^{x2}						0.085 ^{x2}	0.19	2.02	2.213
∑	3.92	3.90	2.13	2.13	2.19	1.97	0.16	1		

Table S7. Bond-valence analysis (*v.u.*) for SMA1971-214 tremolite asbestos, based on the EPMA occupancies calculated following Brese and O'Keeffe[8]. The contribution of the O–H bond has been evaluated according to Ferraris and Ivaldi[9].

(1)	(2)
cm^{-1}	cm^{-1}
1063	1062
1030	1031
950	950
933	932
746	751
674	676
580	-
532	531
518	516
-	438
419	418
395	396
372	373
308	306
253	254
235	234
223	225

Table S8. Raman bands position for tremolite in the region 1200-200 cm^{-1}. (1) = this study. (2) = tremolite from Rinaudo et al.[10].

	Chlorite		Lizardite	
	Wt%	σ	Wt%	σ
SiO_2	31.49	0.33	38.00	0.87
Al_2O_3	16.02	0.01	5.86	1.28
Cr_2O_3	0.05	0.05	0.43	0.25
MnO	1.58	0.05	0.02	0.01
MgO	16.88	0.10	36.51	1.52
CaO	0.19	0.15	0.08	0.02
Na_2O	0.02	0.03	0.01	0.01
K_2O	0.02	0.01	0.01	0.01
NiO	0.01	0.03	0.06	0.01
FeO	23.61	0.38	4.05	0.64
Total	89.86	0.46	85.21	0.59

Table S9. Major element composition of chlorite and lizardite. Data obtained from EPMA analysis

Serpentine discrimination

Serpentine minerals chrysotile, lizardite and antigorite are stable under a wide range of temperatures and pressures[11-13]. Lizardite and chrysotile are stable at low-pressure low-temperature conditions (0–300 °C, < 1.0 GPa), but lizardite is usually more stable than chrysotile[13]. Antigorite is the high-pressure and high-temperature stable serpentine. Both experimental and natural observations show that the lizardite to antigorite transition starts at about 300 °C, with P> 0.7 GPa, with a complete transformation to antigorite near 400 °C[13].

Among the minerals of the serpentine only chrysotile shows a fibrous aspect[11] In addition, chrysotile is poor in Al and Fe, while antigorite and lizardite are enriched in Fe^{+3} and Al[11]. The serpentine found in the SMA1971-214 shows non-fibrous habit (Fig. 3S) and its Al content (> 5.0 wt%) is similar to that of lizardite and antigorite[14] (Table S9). On the assumption that sample SMA1971-214 was developed in a low temperature and pressure regime (see the main text of the manuscript), antigorite can be excluded, and the serpentine in the sample SMA1971-214 can be guessed as lizardite.

Structure refinement of tremolite.

A total of 1768 frames were collected, using φ and ω scan modes, with an exposure time of 30s per frame. Intensity data were integrated and corrected for Lorentz, polarization, background effects, and absorption using the APEX 3 software package[15]. The total exposure time was 14.73 h. The integration of the data using a monoclinic unit cell yielded a total of 12016 reflections to a maximum θ angle of 29.99° (0.71 Å resolution). Scattering curves for neutral atoms were taken from the International Tables for Crystallography[16]. The final cell constants a = 9.8469(6) Å. b = 18.0651(11) Å. c = 5.2795(4) Å. β = 104.803(3)°. V = 907.98(9) $Å^3$, are based upon the refinement of the XYZ-centroids of 7068 reflections above 20 σ(I) with 4.51° < 2θ < 29.99°. Structural model for tremolite as reported by Yang & Evans[17] was assumed as a starting one for structural refinement. Several cycles of isotropic refinement led to R1 = 0.11 confirming the correctness of the structural model. Mixed occupancies by Mg and Fe for $M(1)$, $M(2)$ and $M(3)$ sites and Si and Al for $T(1)$ site were then

refined., with the constrain of full occupancy. Some further cycles of anisotropic refinement allowed then to identify in the Fourier difference maps peaks corresponding to *M*(4') split site, *A* site and *H* site. Following literature studies[17-19] an occupancy by Fe was assumed for *M*(4') site, refining an isotropic displacement parameter and constraining the sum of *M*(4') and *M*(4) occupancies to full occupancy. An occupancy by Na for *A* site was assumed and allowed to vary unconstrained, together with an isotropic displacement parameter. *H* site was assumed as fully occupied, holding fixed its positional parameters derived from the Fourier difference maps, and refining an isotropic displacement parameter.

References

1. Brunelli, D. *et al.* Origin of oceanic ferrodiorites by injection of nelsonitic melts in gabbros at the Vema Lithospheric Section, Mid Atlantic Ridge. *Lithos* **368-369**, 105589 (2020).

2. Cipriani, A. *et al.* A 19 to 17 Ma amagmatic extension event at the Mid-Atlantic Ridge: Ultramafic mylonites from the Vema Lithospheric Section. *Geochem. Geophys. Geosyst.* **10**, 10 (2009).

3. Sarbas, B. & Nohl U. The GEOROC database as part of a growing geoinformatics network in *Geoinformatics 2008-Data to Knowledge* (eds. Brady, S.R., Sinha, A.K., and Gundersen, L.C.) 42-43 (United States Geological Survey, 2008).

4. EathChem. Datasets downloads from https://www.earthchem.org/petdb (2020).

5. Hawthorne F.C. *et al.* Nomenclature of the amphibole supergroup. *Am. Mineral.* **97**, 2031-2048 (2012).

6. Ernst, W.G. & Liu, J. Experimental phase-equilibrium study of Al- and Ti-contents of calcic amphibole in MORB; a semiquantitative thermobarometer. *Am. Mineral.* **83**, 952–969 (1998).

7. Robinson, K., Gibbs, G. V. & Ribbe, P. H. Quadratic elongation: a quantitative measure of distortion in coordination polyhedra. *Science*. **172**, 567-570. (1971).

8. Brese, N. B. & O'Keefe, M. Bond-Valence Parameters for Solids. *Acta Cryst.* **B47**, 192-197 (1991).

9. Ferraris, G. & Ivaldi, G. Bond valence vs bond length in O···O hydrogen bonds. *Acta Cryst.* **B44**, 341-344. (1988).

10. Rinaudo. C., Belluso. E. & Gastaldi. D. Assessment of the use of Raman spectroscopy for the determination of amphibole asbestos. *Mineral. Mag.* **68**, 455-465 (2004).

11. Ballirano, P. *et al.* Crystal structure of mineral fibres in *Mineral Fibres: Crystal Chemistry, Chemical-Physical Properties, Biological Interaction and Toxicity* (ed. Gualtieri, A.F.) 17-64 (European Mineralogical Union, 2017).

12. Evans, B. W. The serpentinite multisystem revisited: chrysotile is metastable. *Int. Geol. Rev.* **46,** 479-506 (2004).

13. Guillot, S., Schwartz, S., Reynard, B., Agard, P. & Prigent, C. Tectonic significance of serpentinites. *Tectonophysics*. **646**, 1-19 (2015).

14. Anthony, J. W., Bideaux, R. A., Bladh, K. W. & Nichols, M. C. *Handbook of Mineralogy* http://www.handbookofmineralogy.org (2020).

15. Bruker AXS Inc. APEX 3 in *Bruker Advanced X-ray Solution* (Bruker AXS Inc., 2016).

16. Wilson, A. J. C. *International Tables for Crystallography. Volume C.* (Kluwer Academic Publishers, 1992).

17. Yang. H. & Evans. B. W. X-ray structure refinements of tremolite at 140 and 295 K: Crystal chemistry and petrologic implications. *Am. Mineral.* **81,** 1117-1125. (1996).

18. Oberti, R., Ungaretti L., Cannillo E., Hawthorne F. C. & Memmi I. Temperature-dependent Al order-disorder in the tetrahedral double chain of *C*2/*m* amphiboles. *Eur. J. Mineral.* **7**, 1049-1063 (1995).

19. Hawthorne. F. C. & Oberti. R. Amphiboles: crystal chemistry. *Rev. Mineral. Geochem.* **67**, 1-54 (2007).

References relating to Figure 4 present in the main text

Tremolite in oceanic tectonic settings

- Sarbas, B. & Nohl U. The GEOROC database as part of a growing geoinformatics network in *Geoinformatics 2008-Data to Knowledge* (eds. Brady, S.R., Sinha, A.K., and Gundersen, L.C.) 42-43 (United States Geological Survey, 2008).

- EathChem. Datasets downloads from https://www.earthchem.org/petdb (2020).

- Cipriani, A. *et al.* A 19 to 17 Ma amagmatic extension event at the Mid-Atlantic Ridge: Ultramafic mylonites from the Vema Lithospheric Section. *Geochem. Geophys. Geosyst.* **10**, 10 (2009).

- Prigent, C., Warren, J. M., Kohli, A. H. & Teyssier, C. Fracture-mediated deep seawater flow and mantle hydration on oceanic transform faults. *Earth Planet. Sci. Lett.* **532**, 115988 (2020).

Tremolite in continental tectonic settings

- Vignaroli, G., Ballirano, P., Belardi, G. & Rossetti, F. Asbestos fibre identification vs. evaluation of asbestos hazard in ophiolitic rock me´langes, a case study from the Ligurian Alps (Italy). *Environ. Earth Sci.* **72**, 3679-3698 (2014).

- Liu, Y. *et al.* Chemical zone of nephrite in Alamas, Xinjiang, China. *Resour. Geol.* **60**, 249–259 (2010).

- Liu, Y., Deng, J., Shi, G., Sun, X. & Yang, L. Geochemistry and petrogenesis of placer nephrite from Hetian, Xinjiang, Northwest China. *Ore Geol. Rev.* **41**, 122–132 (2011).

- Zhang, C., Yu, X., Yang, F., Santosh, M. & Huo, D. Petrology and geochronology of the Yushigou nephrite jade from the North Qilian Orogen, NWChina: Implications for subduction-related processes. *Lithos* **380–381**, 105894 (2021).

- Sanchez, M. S. & Gunter, M. E. Tests of the correlation between composition and morphology of tremolite from Montgomery County, Maryland, USA. *Period. Mineral.* **77**, 15-25 (2008),

- Lahondère, D., Cagnard, F., Wille, G. & Duron, J. Naturally occurring asbestos in an alpine ophiolitic complex (northern Corsica, France). *Environ. Earth. Sci.* **78**, 540 (2019).

- Dichicco, M. C., Paternoster, M., Rizzo, G. & Sinisi, R. Mineralogical asbestos assessment in the southern Apennines (Italy): A review. *Fibers* **7**, 24 (2019).

- Dorling, M. & Zussman, J. Characteristics of asbestiform and non-asbestiform calcic amphiboles. *Lithos* **20**, 469-489 (1987).

- Ballirano. P., Andreozzi. G.B. & Belardi. G. Crystal chemical and structural characterization of fibrous tremolite from Susa Valley, Italy, with comments on potential harmful effects on human health. *Am. Mineral.* **93**. 1349-1355 (2008).

- Pacella. A., Andreozzi. G. B., Ballirano. P. & Gianfagna. A. Crystal chemical and structural characterization of fibrous tremolite from Ala di Stura (Lanzo Valley, Italy). *Period. Mineral.* **77**. 51-62 (2008).

- Pacella, A., Andreozzi, G. B. & Fournier, J. Detailed crystal chemistry and iron topochemistry of asbestos occurring in its natural setting: A first step to understanding its chemical reactivity. *Chem. Geol.* **277**, 197-206. (2010).

- Scambelluri, M., Pettke, T., Rampone E., Godard, M. & Reusser E. Petrology and Trace Element Budgets of High-pressure Peridotites Indicate Subduction Dehydration of Serpentinized Mantle (Cima di Gagnone, Central Alps, Switzerland). *J. Petrol.* **55**, 459–498 (2014).

- Giacobbe, C. *et al.* Synchrotron nano-diffraction study of thermally treated asbestos tremolite from Val d'Ala, Turin (Italy). *Minerals* **8**, 311 (2018).
